# Rare antibody phage isolation and discrimination (RAPID) biopanning enables identification of high-affinity antibodies against challenging targets

**DOI:** 10.1038/s42003-023-05390-0

**Published:** 2023-10-12

**Authors:** Dong hee Chung, Sophie Kong, Nicholas J. Young, Shih-Wei Chuo, Jamie V. Shiah, Emily J. Connelly, Peter J. Rohweder, Alexandra Born, Aashish Manglik, Jennifer R. Grandis, Daniel E. Johnson, Charles S. Craik

**Affiliations:** 1 Department of Pharmaceutical Chemistry, University of California San Francisco, San Francisco, CA 94158 USA; 2 Department of Otolaryngology – Head and Neck Surgery, University of California San Francisco, San Francisco, CA 94158 USA; 3 The Pharmaceutical Sciences and Pharmacogenomics Graduate Program, University of California San Francisco, San Francisco, CA 94158 USA

**Keywords:** Biotechnology, Proteins

## Abstract

In vitro biopanning platforms using synthetic phage display antibody libraries have enabled the identification of antibodies against antigens that were once thought to be beyond the scope of immunization. Applying these methods against challenging targets remains a critical challenge. Here, we present a new biopanning pipeline, RAPID (**R**are **A**ntibody **P**hage **I**solation and **D**iscrimination), for the identification of rare high-affinity antibodies against challenging targets. RAPID biopanning uses fluorescent labeled phage displayed fragment antigen-binding (Fab) antibody libraries for the isolation of high-affinity binders with fluorescent activated sorting. Subsequently, discriminatory hit screening is performed with a biolayer interferometry (BLI) method, BIAS (**B**iolayer **I**nterferometry **A**ntibody **S**creen), where candidate binders are ranked and prioritized according to their estimated kinetic off rates. Previously reported antibodies were used to develop the methodology, and the RAPID biopanning pipeline was applied to three challenging targets (CHIP, Gαq, and CS3D), enabling the identification of high-affinity antibodies.

## Abstract (web summary)

A FACS aided selection process combined with characterization of the selected clones facilitates phage display screening to obtain antibodies against a given target from libraries.

## Introduction

Antibodies (Abs) have and continue to be invaluable tools for biological research^1^. In particular, the need for high-quality antibodies was apparent during the 2020 SARS-CoV-2 pandemic when the rapid identification of therapeutic interventions was critical^2–4^. While immunization has been the traditional method for Ab discovery, biopanning using synthetically displayed Ab libraries (i.e., phage display and yeast surface display) have expanded the field and allowed for non-protein antigens (Ags) such as DNA^5^ and RNA^6^ to be targeted. Furthermore, the in vitro selection and screening nature of the methods enable the identification of Abs in conditions otherwise impossible with immunization (i.e., changes in pH, temperature, oligomeric complex states, ligand states, etc.).

A Standard phage display biopanning methods largely consists of two stages: (1) Iterative rounds of selection and amplification of displayed Abs bound to immobilized Ag to achieve an enriched population of higher-affinity binders; (2) Random screening of enriched pools of displayed Abs for the identification of high-affinity Ab clones (hits). While phage display based biopanning has proven to be a powerful technique, it remains challenging to identify, and characterize selective, high-affinity Abs when their prevalence is extremely low. Examples of these Ags include those that exhibit low antigenicity, or conformational heterogeneity. Poor enrichment towards Ag-Ab binding allows additional factors such as amplification discrepancies of phagemid-containing *E. coli*, and inconsistent display propensities of phage, to undermine the enrichment process resulting in an overall depletion of promising binders with continued rounds of selection. Consequently, significantly larger number of clones need to be screened, and as this process largely relies on stochastic clone picking, rare high-affinity binders can be left unexamined.

These challenges have been met with some effective solutions such as improvements in synthetic Ab library design^7^, hybrid selection techniques by employing yeast surface display (YSD), coupled with fluorescence-activated cell sorting (FACS)^8^, and functional selection-based methods for identifying candidate clones^9^. Yet, further improvements in the current platforms for efficient recombinant Ab discovery are crucial for continued expansion of the field.

**Fig. 1:**
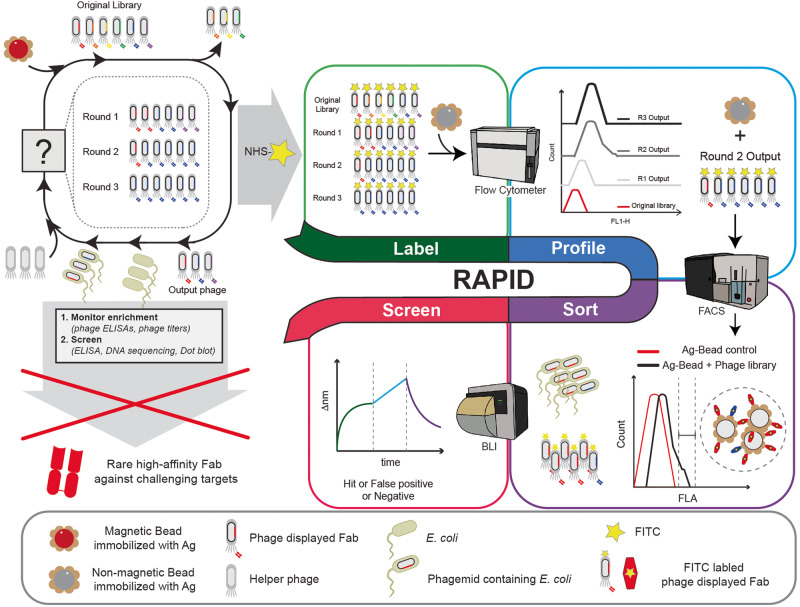
Schematic of RAPID biopanning for the identification of rare high-affinity binders against challenging targets. RAPID biopanning follows a Label-Profile-Sort-Screen pipeline. First, phage libraires from rounds of biopanning are individually fluorescein isothiocyanate (FITC, yellow star) labeled and each incubated with antigen-beads (beige antigen, gray bead). Subsequent analysis with flow cytometry profiles the progression of the biopanning campaign and accurately identifies the most enriched round for antigen-antibody binding. For phage displayed Fab (ph-Fab) libraries that exhibit extremely low frequencies of high-affinity antibodies against antigens, fluorescent activated sorting is performed to isolate specific populations of ph-Fabs that contain higher frequencies of high-affinity binders. A biolayer interferometry (BLI) method is subsequently employed to rapidly screen candidate clones in a discriminatory matter, thereby prioritizing promising hits for further investigations. For comparison, standard biopanning using magnetic beads (beige antigen, red bead) is depicted in the upper left corner. The images of the FACS, flow, and BLI were hand traced by the authors using procreate from images of the instruments.

Here we provide a phage display pipeline, RAPID (**R**are **A**ntibody **P**hage **I**solation and **D**iscrimination), for identifying rare high-affinity fragment antigen-binding (Fab) Abs against challenging targets using a recombinant phage displayed (ph-Fab) library (Fig. 1). The RAPID biopanning pipeline was developed to selectively isolate the most promising population of binders and screen in a discriminatory fashion to allow for a streamlined method to identify high-affinity Fabs. This is enabled by using fluorescent labeled ph-Fab libraries, and a BLI (**B**io**l**ayer **i**nterferometry) method called BIAS (**B**iolayer **I**nterferometry **A**ntibody **S**creen) is used for discriminatory screening of candidate clones.

RAPID biopanning combines the advantages of large phage display libraries (up to 10^10^) with the quantitative screening afforded by the FACS methodology commonly described with YSD^10^. Since the size of phage (~0.9 μm) precludes its use in cytometer detection, we instead used Ag immobilized beads for cytometer detection and quantitation of the fluorescence signal is performed on populations of labeled ph-Fab bound to Ag-beads. In addition, RAPID is not material intensive with only ~20 ug of Ag required for both fluorescent sorting and BIAS experiments and can therefore be used for targets that are difficult to acquire reagent quantities of. Overall, the RAPID pipeline is widely applicable and allows for a highly efficient strategy for the identification of rare high-affinity binders.

Here, we first demonstrate the methodology of each step of RAPID using a set of previously reported antibodies that bind ARS1620, a preclinical, covalent KRas G12C inhibitor^11^. Additionally, biopanning campaigns on three challenging targets are described as examples to highlight the power of RAPID biopanning. **C**arboxyl terminus of **H**sp-70 **i**nteracting **p**rotein (CHIP) is a E3 ubiquitin ligase target that is highly conformationally diverse and lacks conformationally selective Abs for biological studies. Gαq is one of many different heterotrimeric G protein subunits and lacks selective Ab reagents for biological and structural studies. **C**yclic **S**TAT**3 D**ecoy (CS3D) is a double stranded DNA drug candidate, and there is limited precedence for the description of recombinant Abs that bind to dsDNA antigens.

## Results

### The Rare Antibody Phage Isolation and Discrimination (RAPID) pipeline

The RAPID biopanning pipeline comprises four steps: (1) Fluorescent labeling of ph-Fab libraries for the detection and quantification of ph-Fab-Ag-bead complexes; (2) Biopanning campaign enrichment profiling with flow cytometry for the accurate determination of Ag-Fab enrichment; (3) Isolation of high-affinity binder populations with fluorescence activated sorting; and (4) Discriminatory hit screening of candidate clones with **B**iolayer **I**nterferometry **A**ntibody **S**creen (BIAS).

### NHS-FITC labeling of ph-Fab enables quantitative detection of Fab-Ag binding

**Fig. 2:**
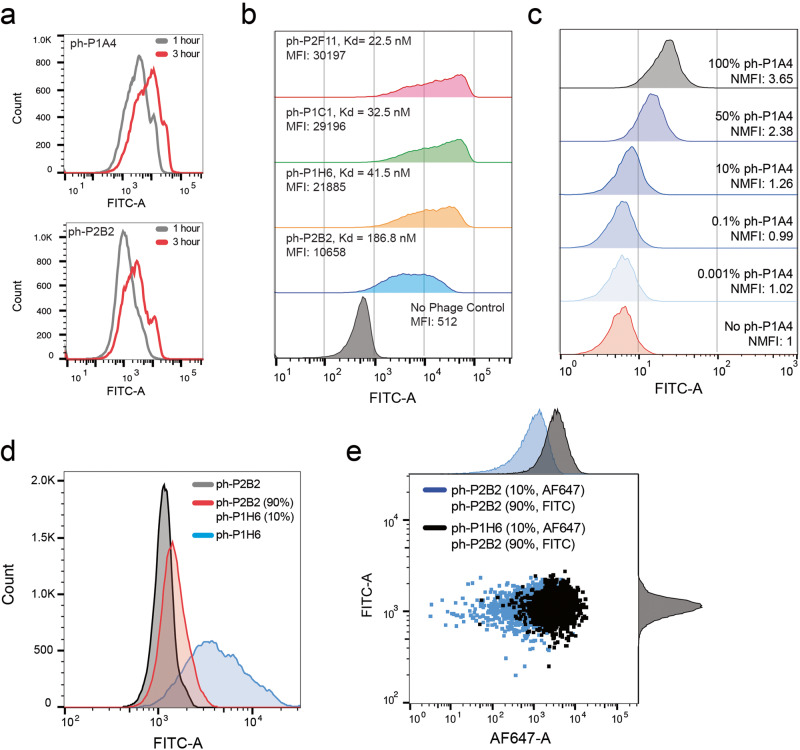
Flow cytometry analysis of fluorescent labeled phage displayed Fabs (ph-Fabs) bound to ARS1620-V7 immobilized beads. **a** ph-Fabs bound to ARS1620-V7-beads with varying incubation times. ph-P1A4 and ph-P2B2 were incubated for a total of 1 h and 3 h each. An increase in fluorescent intensity is observed beyond the 1-hour mark, indicating that the binding is not fully saturated even after 1 h of incubation. **b** Flow cytometry analysis of ph-Fabs of varying affinities bound to ARS1620-V7-beads. Fluorescent distributions of beads bound to ph-P2B2, ph-P1H6, ph-P1C1, and ph-P2F11 show MFI trends that agree with the ph-Fab affinities. **c** Flow cytometry analysis of increasing ph-P1A4 concentration shows an increase in normalized MFI with an observable shift from flow cytometry starting at 10 percent ph-P1A4 (**d**) Overlay of flow cytometry analysis of ph-P2B2, ph-P1H6, and a ph-P2B2, ph-P1H6 mix (90%/10%) bound to ARS1720-V7-beads. (e) Flow cytometry analysis of fluorescein isothiocyanate (FITC) and Alexa Fluor 647 (AF647) labeled mixture of ph-P2B2 and ph-P1H6. Pure ph-P2B2 labeled with both FITC and AF647 was used as a control. Histograms of the dot blot distributions are also shown.

For the RAPID pipeline to be a feasible protocol, fluorescence from Ag-Bead-phage complexes needs to directly correlate to the number of ph-Fabs bound per Ag-Bead. To ensure that fluorescent intensities correlate to the ph-Fab affinity, we optimized the method with four unique ph-Fabs (ph-P1H6, ph-P1C1, ph-P2F11, and phP2B2) that bind to a common Ag, an ARS1620-labeled KRas G12C peptide VVVGACGVGK (ARS1620-V7) (Fig. 2). The duration of ph-Fab incubation with Ag-beads was examined to avoid the over-saturation of Ag-beads with bound ph-Fabs.

Using two FITC-labeled ph-Fabs with varying affinities (ph-P2B2 and ph-P1A4), an incubation period of 1 h generated a fluorescent signal that was sufficiently high. An additional 2 h of incubation resulted in an increase in fluorescent intensity, indicating that the Ag-beads were not saturated after the initial 1-hour incubation (Fig. 2a).

The density of Ag molecules immobilized on beads was also considered to achieve a 1:1 binding mode to ph-Fab, which would ensure that the fluorescent signal observed on each bead accurately reflected the potential maximal quantitative number of ph-Fabs bound. From immobilizing biotin-FITC to streptavidin-beads, biotin-FITC immobilization saturated near a final concentration of 0.1 μM, and less than half of the maximum amount was shown to immobilize at 0.01 μM according to the normalized MFI (Supplementary Fig 1). An antigen concentration of 0.01 μM was used for all subsequent RAPID flow cytometry and FACS experiments.

Applying these conditions, different ph-Fabs were FITC labeled separately and incubated individually with Ag-Beads (Fig. 2b). We observed good agreement between the fluorescent intensity and the affinity of the displayed Fab where a ~ 3-fold difference in MFI is observed between the lowest-affinity binder (ph-P2B2, *K*_d_ = 186.8 nM) and the highest-affinity binder (ph-P2F11, *K*_d_ = 22.5 nM). Furthermore, when examining ph-Fabs with similar affinities (ph-P1H6, *K*_d_ = 41.5 nM, ph-P1C1 *K*_d_ = 32.5 nM, ph-P2F11 *K*_d_ = 22.5 nM), consistent patterns emerge between the displayed *K*_d_ values and the MFI values. This agreement indicates that NHS labeling of ph-Fabs is indeed a reliable and robust technique for fluorescently labeling M13 phage, facilitating their detection in flow cytometry and FACS.

### RAPID flow cytometry for profiling the enrichment of a biopanning campaign

Having established that the MFI correlates to the binding affinity of ph-Fabs, we applied these flow cytometry methods for the characterization of heterogenous ph-Fab populations meant to model those isolated during a biopanning campaign. Mixtures of ph-Fab containing varying ratios of M13 phage and ph-P1A4 were employed to emulate progressive rounds of biopanning, wherein a high-affinity binder (ph-P1A4) is gradually enriched within a pool predominantly comprised of nonbinders (M13 phage). These mixtures were then individually FITC labeled, and flow cytometry analysis was performed with ARS1620 bound beads. A normalized MFI of 1.26 is exhibited with 10% ph-P1A4 when compared to the 0% ph-P1A4 sample (Fig. 2c). Normalized MFI values for a 50% and 100% were 2.38 and 3.65 respectively, confirming that enriched populations have higher fluorescent signal. The range of normalized MFI values act as standards in determining future approximate hit rates of a library of ph-Fab with similar kinetic properties as ph-P1A4 (*K*_d_ ~ 24 nM).

Similarly, by FITC labeling ph-Fab libraries from biopanning campaigns and analyzing by flow cytometry, an accurate, and quantitative distribution of ph-Fab bound to Ag-beads can be observed which is not achievable by previous techniques (i.e., phage ELISA). By applying this approach to consecutive rounds of biopanning within a single campaign, the distribution of binding can be monitored over time (Supplementary Fig 2), allowing the identification of biopanning rounds enriched for binders. The normalized median fluorescent intensity (NMFI) indicates the average affinity of bound ph-Fab population. Additionally, assuming the NMFI of two different populations are similar, the standard deviation (SD) represents the range of affinities of individual binders. By combining this information, dominant factors that influenced the enrichment process during the biopanning campaign can be determined (Supplementary Fig 2). From biopanning profiling data, the round which is the most enriched for Ag-Ab binding can be directly identified and prioritized for screening with BIAS.

### RAPID FACS allows for the isolation of ph-Fab populations that are highly enriched for Ag binding

For cases where significant global Ag-Ab enrichment is not observed, fluorescent bead populations can be sorted using FACS (Fig. 1). A key feature of the RAPID pipeline is the isolation of Ag-Beads exhibiting higher fluorescent signals which harbor bound ph-Fab populations that are enriched with high-affinity binders, which would otherwise exist at low frequencies within the initial binder pool.

To confirm this, a mixture of a high and low-affinity ph-Fab binders was generated to represent ph-Fab libraries containing rare high-affinity binders in low frequencies (Fig. 2d). The fluorescent distribution of Ag-Beads agrees with the binding affinity of the bound ph-Fabs. For ph-P2B2 (*K*_d_ =186.8 nM, MFI = 1147, SD = 295), the distribution demonstrates an MFI with a low SD, indicating a more uniform distribution of ph-Fabs. Conversely, with ph-P1H6 (*K*_d_ = 41.5 nM, MFI = 3901, SD = 4117), the distribution displays a higher MFI and a higher SD, suggesting a less even distribution of ph-Fabs. This observation can be rationalized by considering that weaker binders have a greater capacity to redistribute their populations compared to stronger binders in a pre-equilibrium system. This is consistent with the results we observe when a mixture of ph-P2B2 (90%) and ph-P1H6 (10%) is examined as the distribution exhibits an intermediate MFI and SD, relative to the pure samples (MFI = 1446, SD = 552).

Furthermore, we investigated the correlation between higher fluorescent signals and enriched populations of high-affinity ph-Fabs by generating a mixture of ph-Fab comprising a majority population (90%) and a minority population (10%) that were labeled separately with FITC and AF647 respectively, and thereafter combined (Fig. 2e). A low or high-affinity binder (ph-P2B2, *K*_d_ = 186.8 nM, ph-P1H6, *K*_d_ = 41.5 nM) was used as the minority population, while the majority population was kept constant as the low-affinity ph-P2B2. In parallel, both mixtures were incubated with Ag-beads and FITC (majority population) and AF647 (minority population) fluorescent distributions were analyzed by flow cytometry.

The distribution of the majority population is near identical between the two mixtures according to their FITC-MFI (ph-P1H6 mixture MFI = 1118, ph-P2B2 mixture MFI = 1095), while an increase in fluorescent intensity and SD is observed for the ph-P1H6 containing mixture, shown by the increase in AF647-MIF and AF647-SD (ph-P1H6 mixture MFI = 3327, ph-P2B2 mixture MFI = 987), (ph-P1H6 mixture SD = 2338, ph-P2B2 mixture SD = 932). This indicates that the binding events of low or high-affinity binders occur independently and retain their respective binding distribution characteristics. Specifically, Ag-Beads are evenly occupied by the low-affinity binder, while simultaneously high-affinity binders are unevenly distributed. As a result, beads with higher fluorescent signals demonstrate increased intensities, primarily due to the greater abundance of higher affinity binders, rather than the redistribution of the lower affinity binders. Our results confirm that Ag-Beads exhibiting higher fluorescent signals are indeed associated with greater enriched populations of higher-affinity ph-Fabs, and isolation of these populations can be achieved with FACS.

### Biolayer interferometry antibody screen (BIAS) for discriminatory screening of candidate binders

Despite the capacity of both RAPID flow cytometry and FACS to facilitate the identification and isolation of considerably enriched populations of ph-Fab bound to Ag-Beads, it does not guarantee the exclusion of weaker binders from the isolated pool. To address this, BIAS was developed for the discriminatory screening of candidate Abs by analyzing the real-time binding of individual candidate Abs directly from crude periplasmic extract (PPE) samples using BLI. This is distinct from commonly used methods for candidate binder screening (i.e., dot blot, ELISA, DNA sequencing) where hits are not fully distinguishable based on their binding properties. Additionally, BIAS only requires a maximum ~3 μg of Ag to assay 95 candidate clones which can be screened within 3 h, which makes this technique amenable to targets that are difficult to express/purify. BIAS can also be applied to a variety of different assay conditions (i.e., temperature, buffer, etc.) and in addition to binding properties of candidate clones, candidate binders that exhibit severely low expression levels can also be identified for deprioritization.

**Fig. 3:**
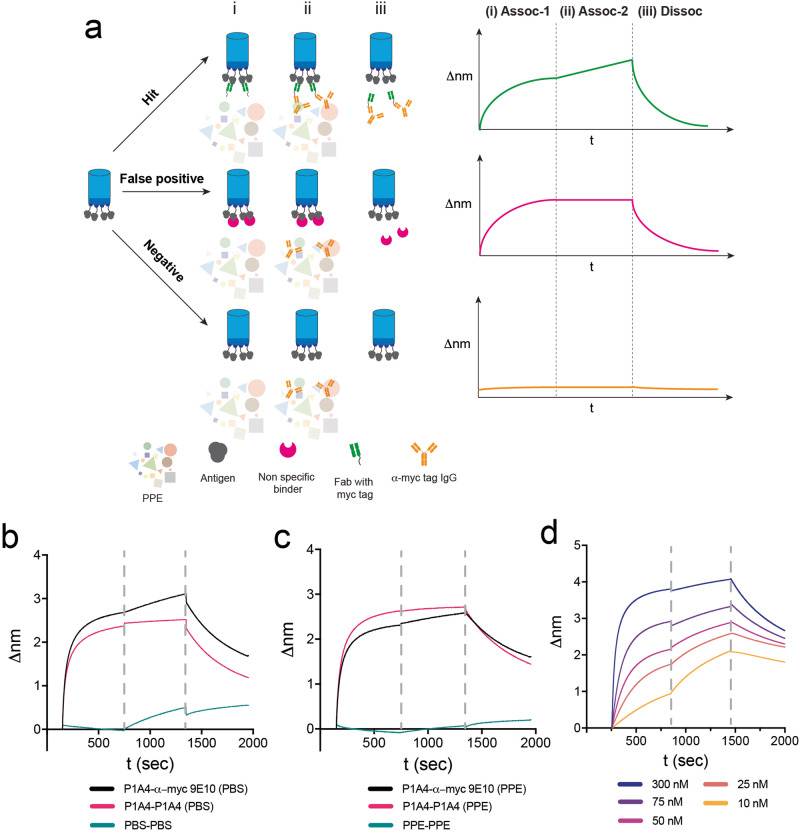
Biolayer interferometry antibody screen (BIAS) scheme and control experiments. **a** BIAS scheme. Representative biolayer interferometry (BLI) sensograms of each distinct outcome (Hit, False positive, Negative) are shown. (i) Association-1: Biological tips bound with Ag are transferred to crude PPEs induced for expression, (ii) Association-2: tips are transferred to wells containing identical PPE sample from Association-1 + anti-myc IgG, (iii) Dissociation: tips from (ii) are transferred to wells with no PPE and no IgG. The Association-2 step distinguishes hits versus false positives. **b**–**d** BLI sensograms of BIAS performed with ARS1620 and P1A4 spiked samples. **b** P1A4 (250 nM) spiked in PBS. A signature linear curve is observed in Assco-2 where the anti-myc antibody (9E10) is present, while this is not observed with the control with no 9E10. **c** P1A4 (250 nM) spiked in TG1-PPE. Results are similar as (**c**). **d** Concentration series of P1A4 spiked in TG1-PPE (10–300 nM).

BIAS consists of three steps (Fig. 3a). (i) Association-1 (Assoc-1): Ag loaded tips are transferred to crude PPEs containing expressed Fabs allowing Fabs to associate to the Ag, (ii) Association-2 (Assoc-2): tips are transferred to wells containing identical PPE + anti-tag IgG, where the anti-tag IgG can bind to the Fab (iii) Dissociation (Dissoc): tips are transferred to wells containing buffer where Fabs are dissociated. BIAS is able to identify true positive binders from a characteristic association curve observed in the Assoc-2 step which is absent for false positive clones. For our purposes, our entire ph-Fab library includes a myc-tag, therefore, we employed the anti-myc IgG, 9E10, for our Assoc-2 step. This Ab has been reported to be highly specific where one amino acid change has shown to disrupt binding^12^ therefore binding in Assoc-2 is a direct readout of tagged Fab binding.

An in-house developed script, **B**IAS **A**lgorithm **T**riaging **C**onfirmed **H**its (BATCH), individually analyzes the binding curves and categorizes each individual clone into hits, false positives, and negatives (Supplementary Fig 3). For hits, we use predicted *k*_off_s as the distinguishing factor for rank ordering binders as *k*_on_ predictions are inaccurate due to the presence of inconsistent PPE in the Assoc-1 step (Supplementary Fig 4).

### Characterizing BIAS with P1A4 and ARS1620

To establish proof of concept of the BIAS assay, we used the Fab-Ag pair, P1A4-ARS1620 (*k*_off_ = 2.07 × 10^−3 ^s^−1^, *K*_d_ = 25.1 nM). P1A4 was tested against an ARS1620 labeled peptide at 250 nM (both Assoc-1 and Assoc-2) where the anti-myc IgG (9E10) was either included or absent in Assoc-2. Solutions of P1A4 spiked into both PBS and crude PPE derived from TG1 cells (*E. coli*) were tested in parallel to determine whether the method could tolerate the presence of the highly heterogenous PPE. An association curve is clearly observed in the Assoc-2 phase in samples that contains the secondary IgG, 9E10, when compared to the samples that do not (Fig. 3b, c) confirming that true positives are identifiable in the Assoc-2 step. Predicted kinetic parameters including *k*_off_s from BATCH yields near identical values to reported *k*_off_s tested in vitro indicating that the addition of the Assoc-2 step or the PPE environment does not interfere with kinetic predictions (supplementary table 1).

Varying concentrations of P1A4(10–300 nM) were also tested to establish concentration dependencies of the assay (Fig. 3d). A series of concentrations of P1A4 spiked in PPE shows an increase in true Assoc-2 slopes as concentration approaches *K*_d_ (Supplementary Table 2). Where the Assoc-1 step is not fully saturated, and the P1A4 sample concentrations of are at non-steady-state conditions, *k*_off_ predictions are less accurate. To address this, we developed BATCH to flag hits that show low expression or no saturation in Assoc-1 to indicated inaccuracies in predicted *k*_off_ values for the user’s discretion.

### RAPID biopanning enables the identification of rare antibodies against CHIP

**C**arboxyl terminus of **H**sp-70 **i**nteracting **p**rotein (CHIP) is an E3 ubiquitin ligase canonically known to interact with heat shock protein 70 (Hsp70) and heat shock protein 90 (Hsp90), leading to the ubiquitination of misfolded clients as well as regulation of chaperone turnover. Recent work demonstrated that CHIP has substrate specificity extending beyond Hsp70/90 and predicted interactions suggest CHIP may have additional Hsp-independent roles in proteostasis and disease states^13^. To enable further biological studies, we sought to develop Abs against CHIP beyond the available substrate-binding inhibitors^13,14^. CHIP is a challenging biopanning target due to its homodimeric structure and conformational flexibility, and therefore we employed RAPID biopanning.

**Fig. 4:**
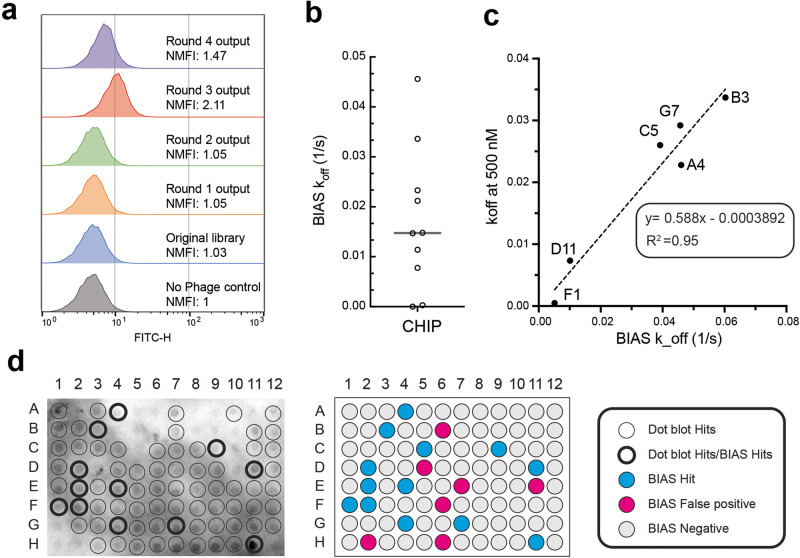
Rare antibody phage isolation and discrimination (RAPID) biopanning of **C**arboxyl terminus of **H**sp-70 **i**nteracting **p**rotein (CHIP). **a** RAPID flow cytometry of Rounds 1–4. Round 3 shows the highest NMFI of 2.11. **b** Comparison of Biolayer interferometry antibody screen (BIAS) *k*_off_ and biolayer interferometry (BLI) measured *k*_off_ at 500 nM. **c** BIAS *k*_off_ of candidate hits. **d** Candidate hit screening using dot blot and BIAS. Dot blot hits are circled in black where hits that overlap with BIAS hits are circled with a thicker boarder. BIAS Hits, False positives, and Negatives are shown according to the legend.

Four rounds of standard biopanning were employed against CHIP with increased stringency each round. Subsequently, RAPID flow cytometry was performed by individually FITC labeling each output round. Flow cytometry data shows an increase in normalized MFI from round 2 through 3, and a significant drop is observed at round 4 (Fig. 4a). The most enriched round (round 3) shows a normalized MFI of 2.11 which correlates to a reasonably high hit rate between 10 and 50 percent of a high affinity binder (~*K*_d_ = 25 nM) according to previous control experiments with P1A4 (Fig. 2c). As flow cytometry data suggests strong Fab-Ag binding enrichment had occurred, FACS was deemed unnecessary, and BIAS was performed directly.

95 Individual colonies were picked from round 3, and from PPE samples, 18 hits were identified by BIAS, and BATCH calculated *k*_off_s showing a wide range of values (9.40 × 10^6^ − 5.44 × 10^2^ s^−1^) (Fig. 4b, Supplementary Fig 7, Supplementary Table 3). Candidate hit Fabs that were recombinantly expressed and biochemically characterized exhibited similar trends of *k*_off_s compared to those predicted by BIAS (*R*^2^ = 0.95), and their values were within ~2 fold of each other (Fig. 4c). In parallel dot blots and ELISAs were performed to compare BIAS with standard screening methods. Dot blot experiments resulted in 76 hits (80% hit rate) in comparison to 18 hits identified from BIAS (Fig. 4d). After exhaustive optimizations, no hits were identified using ELISAs. The biopanning campaign of CHIP demonstrates the power of RAPID biopanning as a method for accurately identifying the most enriched round, determining dominant factors of enrichment, and predicting hit rates of specific rounds of candidate hit screening. Notably, in this example, BIAS is shown to be a critical step in identifying true functional candidate hits and prioritizing potential binders for detailed biochemical characterizations.

### RAPID identifies rare binders against Gαq

Gαq is a ubiquitous heterotrimeric G protein subunit important in many G protein-coupled receptor signaling cascades. Like other G proteins, the conformation of Gαq is regulated by a guanine nucleotide. While Gαq is normally activated in diverse physiological states, several mutations lead to inappropriate activation of Gαq, leading to challenging diseases like uveal melanoma^15^ or Sturge Weber syndrome^16^. To understand how mutations in Gαq lead to activation, we sought to develop antibody fragments that would enable structural studies. Gαq is a challenging target due to its known dynamic conformational flexibility.

**Fig. 5:**
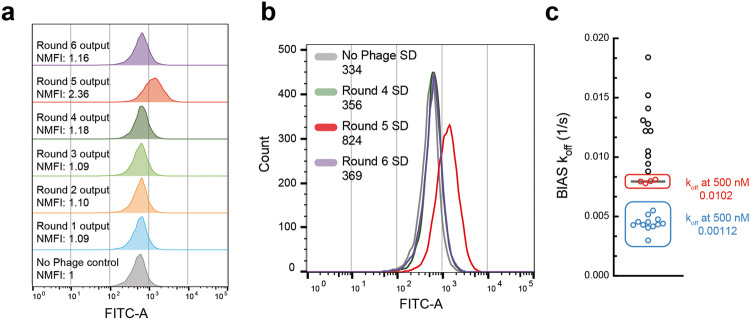
Rare antibody phage isolation and discrimination (RAPID) biopanning of Gαq. **a** RAPID flow cytometry of Rounds 1–6. Round 5 shows the highest normalized median fluorescent intensity (NMFI) of 2.36. **b** Overlay of RAPID flow cytometry of round 4–6 and a no phage control. An increase in standard deviation (SD) of the fluorescent distribution is observed in round 5. **c** Biolayer interferometry antibody screen (BIAS) *k*_off_ of candidate hits. Blue data points represent identical values, while red data points also represent identical values.

Six rounds of biopanning were employed against Gαq using the standard method, and RAPID flow cytometry was used to monitor the enrichment progression (Fig. 5a). A late enrichment is observed where the maximum NMFI (2.36) is reached in round 5. Interestingly, we also observe an increase in SD which would suggest that the high-affinity binders also exhibit superior expression/display propensities (Fig. 5b, Supplementary Fig 2). In line with the findings from the CHIP biopanning campaign, where significant enrichment was achieved, RAPID FACS was not necessary.

BIAS was employed to 95 clones from round 5, where 27 hits were identified (28% hit rate) (Supplementary Fig 7, Supplementary Table 3). The clones occupying the top 12 ranks were determined to be identical. Additionally, independent of these, clones ranked 13 to 16 were also found to be identical (Fig. 5c). Identical clones exhibited similar BIAS *k*_off_ values, and as demonstrated by the CHIP biopanning campaign, the candidate hit Fabs, which were biochemically characterized, displayed comparable *k*_off_ values to those predicted by BIAS. Seven hits were predicted to be low expressors (flagged with*) and are ranked low on the BIAS rankings. This agrees with the RAPID flow cytometry results, where it was predicted that higher-affinity binders would also exhibit higher expression levels.

### RAPID biopanning identifies rare binders against CS3D

**C**yclic **S**TAT**3 d**ecoy (**CS3D**) is a dsDNA decoy that targets STAT3 and inhibits the expression of downstream STAT3-induced genes^17,18^. To date, only a few recombinant Abs have been developed against nucleic acid targets, with most of them targeting unique structural motifs^5,6^. We present the identification of Fabs against CS3D, a 15-mer dsDNA target with no distinct structural motifs, as an example of a challenging target for which limited Ab discovery precedence exists. Both standard biopanning and RAPID biopanning were performed in parallel to provide a head-to-head comparison of the two methods.

With the standard method, a total of six rounds of biopanning was performed with increasing stringency of washes and decreasing amounts of Ag per round. 95 colonies were randomly picked each round 4–6 (285 clones total) for candidate hit screening using dot blots. Four unique Fabs (3B11, 3B12, 3C8, and 4E4) were identified from 38 randomly sequenced clones, where three of the Fabs (3B11, 3B12, and 3C8) differed by only one amino acid indicating that a strong enrichment had occurred for a specific family of related clones over others throughout the rounds. Purified Fabs exhibited weak binding against CS3D with *k*_off_ values ranging from 7.64 ×10^−2^ – 2.77 × 10^−1^ s^−1^ at 2 μM Fab concentrations (supplementary table 4).

To thoroughly examine candidate clones that were picked from the standard method campaign, BIAS was employed to the same clones picked previously that were randomly sequenced. BIAS resulted in a hit rate of 230 out of 285 clones (81% hit rate) (supplementary table 5). However, when the top 10 ranked candidates from each round where sequenced, they were revealed to be the same low-affinity clones that were identified previously (supplementary Table 6), with two additional weak binders 2C11 (*k*_off_, 4.19 × 10^−1 ^s^−1^) and 2B12 (*k*_off_, 3.7 × 10^−1 ^s^−1^) being identified.

**Fig. 6:**
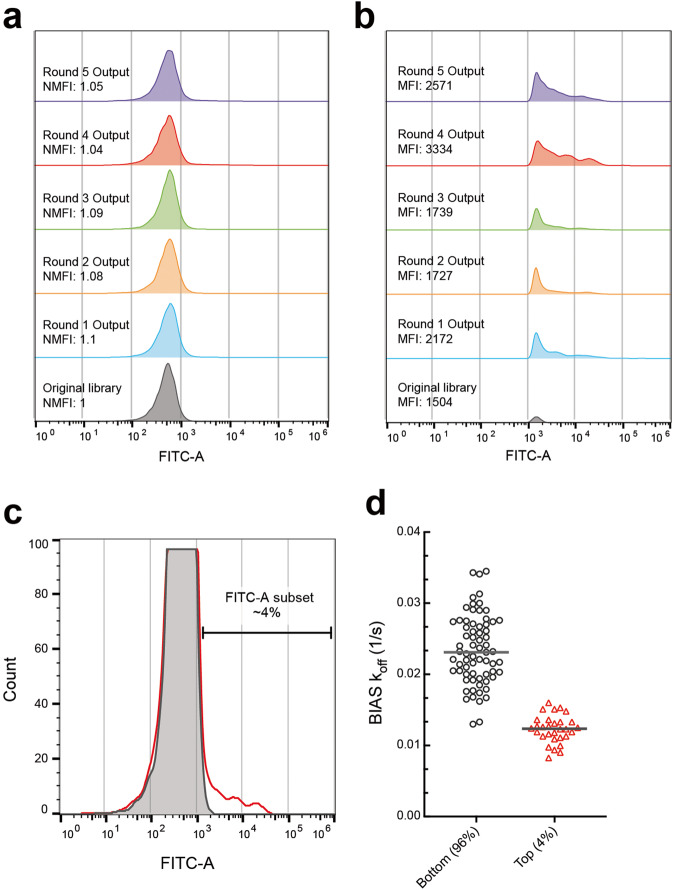
Rare antibody phage isolation and discrimination (RAPID) biopanning of cyclic STAT3 decoy (CS3D). **a** RAPID flow cytometry of biopanning campaign rounds 1–5. No significant increase in normalized median fluorescent intensity (NMFI) is observed. **b** Zoom in of (**a**) populations where FITC-A > 10^3^. Maximum NMFI is exhibited in round 4. **c** Overlay of RAPID flow cytometry of round 4 (red, unfilled) and the original library control (gray, filled), **d** Biolayer interferometry antibody screen (BIAS) *k*_off_s of the top 4% and the bottom 96% of round 4 randomly chosen clones. The top ~4% sorted population contains ~2-fold lower BIAS *k*_off_s suggesting more promising binders exist in the sorted versus unsorted population of phage.

RAPID biopanning was employed to identify higher affinity binders which was not achievable using the standard method. RAPID flow cytometry results showed that nearly no enrichment of binding had occurred during the campaign, as shown with no significant changes of normalized MFI in any of the rounds (Fig. 6a). However, a higher fluorescent signal that does not significantly overlap with the control distribution was observed which showed a maximum MFI at round 4 (Fig. 6b). RAPID FACS was employed to isolate this population.

Beads from the round 4 output (top ~4% fluorescent intensity) were sorted along with the beads from the majority population (bottom ~96%) and both populations were used to infect TG1 (*E. coli)* cells (Fig. 6c). BIAS was performed separately against both populations where ~2-fold lower average BIAS *k*_off_s were observed in the top ~4 percent population hits compared to the bottom ~96 percent population hits (*P* < 0.001), suggesting that the top ~4 percent population contained ph-Fab that have more favorable kinetic properties (Fig. 6d). BIAS hits of the top ~4 percent sorted population were sequenced, and a new candidate binder, SP1-B3, was identified, along with identical clones of previously identified low-affinity binders. SP1-B3 exhibited 2.11 × 10^−2 ^s^−1^ *k*_off_ which is ~3-fold lower than the previously identified Fab with the lowest *k*_off,_ and over ~20- fold lower than the Fab with the highest *k*_off_ that was most frequently identified (supplementary table 4). It is important to note that Fabs that were identified without RAPID biopanning exhibited significantly higher expression levels, as high as 27-fold (supplementary table 4). As it has been well reported that the expression levels of displayed protein correlate with display propensities^19^ it is reasonable to conclude that display propensity bias was a strong factor during the biopanning campaign of CS3D, which led to the enrichment of weakly binding but high expressing ph-Fabs. The use of RAPID in this biopanning campaign enabled the direct isolation of a lower frequency, high-affinity binder population, which resulted in the discovery of superior binding Abs, that otherwise would have not been discovered with the standard method.

## Discussion

The efficient identification of rare high-affinity Abs against challenging targets (i.e., low thermal stability, conformationally diverse, low expression of target, low antigenicity, low solubility, etc.) continues to be a major difficulty in the Ab discovery field. For phage display-based biopanning methods, challenging targets often exhibit weak Ab-Ag enrichment due to the low prevalence of high-affinity binders, and additional factors such as growth biases and inconsistent protein display propensities can affect, and even dominate the enrichment process. This ultimately leads to high-affinity binders never fully enriching and becoming difficult to identify, given the low probability of identification from common stochastic hit-picking screening methods.

The RAPID biopanning method was specifically designed as a solution for identifying rare high-affinity Abs against challenging targets using phage display. Where previous standard methods employ an approach where the total population of enriched displayed Abs are screened in a non-discriminatory fashion, RAPID biopaninng identifies a selective population of high-affinity binders that are subsequently screened in a discriminatory manner. This was achieved by (1) accurately identifying the most enriched population of ph-Fab, (2) increasing the prevalence of low frequency, high-affinity ph-Fabs by fluorescent activated sorting, and (3) rapidly screening candidate hits in a discriminatory matter to prioritize in-depth biochemical characterizations of promising binders. We developed a simple method for fluorescent labeling ph-Fab for quantitative measurements of ph-Fab binding to Ag immobilized beads and a BLI method, BIAS, was developed for rapid real-time analysis of candidate binders. Ultimately, RAPID biopanning follows a Label-Profile-Sort-Screen pipeline and has been applied to three targets, CHIP, Gp, and CS3D where rare high-affinity binders were identified.

Robust labeling of ph-Fab was achieved using NHS-FITC. The labeling reaction is simple, fast, reliable, and effective for the quantitative measurement of ph-Fab bound to Ag-Beads in flow cytometry. Most importantly, FITC labeling does not disrupt the binding of displayed Fab to Ag, nor does it exhibit any significant variabilities in labeling between different ph-Fabs.

By individually FITC labeling ph-Fab libraries from a single biopanning campaign and analyzing bound ph-Fab on Ag-Beads on flow cytometry, the enrichment progression of the campaign can be profiled. The progression of the global distribution of bound ph-Fab from subsequent rounds of biopanning can indicate dominant factors that affected enrichment (i.e., Fab-Ag binding, growth rates of phagemid containing *E. coli*, Fab display propensities). This is superior to phage ELISAs and calculations of phage titers of output ph-Fabs as these methods yield a single measurement of bound phage as opposed to a global distribution.

For campaigns that struggle to identify higher-affinity binders, FACS of higher fluorescent populations can be an effective solution, as beads that exhibit higher fluorescent intensities contain greater enriched populations of higher-affinity binders. The frequency and variation in the affinity of the higher-affinity binder can result in either a broader fluorescence distribution or a discernible population in FACS, as observed in the CS3D biopanning campaign. As shown with the CS3D biopanning campaign, a rare higher affinity binder, SP1-B3, was identified by isolating the ph-Fab-Ag-bead complexes exhibiting higher fluorescent signal by FACS. It is worth noting that previous efforts of standard magnetic bead biopanning followed by random clone picking or even applying the more stringent BIAS screening to these clones failed to identify new higher-affinity binders, indicating that the pool of ph-Fab examined itself had severely low frequencies of higher-affinity ph-Fabs. In the case for the CS3D campaign, the significantly lower expression levels of SP1-B3 (as much as ~20-fold less) compared to the weaker binders suggests that, at least in part, Fab display propensities played a role in the enrichment process. The example of the CS3D biopanning campaign highlights the use of fluorescently labeled ph-Fab libraries coupled with FACS, where rare high-affinity binders can be retrieved from a large pool of weaker binders, previously enriched for factors other than Fab-Ag binding.

It is crucial to the outcome of the antibody discovery campaign to correctly prioritize candidate binders for in-depth biochemical characterization as time and resources are practically limited in a lab setting. To this point, a discriminatory hit screening method, BIAS, was developed to use Abs from crude extracts to measure real-time binding to immobilized Ags using BLI. The raw data is analyzed with an in-house developed script, BATCH, and clones are categorized and rank ordered based on their kinetic off-rates. The discriminatory identification of clones allows for the prioritization of more promising candidates and further investigation and characterizations can be done in line with the BIAS rankings. As shown with the CHIP biopanning campaign, BIAS is more discriminatory compared to conventional screening methods such as dot blots and provides more in-depth details of candidate clones compared to ELISAs (i.e., Fab expression levels, *k*_off_ values, etc.). This is demonstrated by the most promising candidate binder, F1, showing no obvious indication of being a promising candidate from dot blot results (Fig. 4d). BIAS hits from the CHIP campaign show similar kinetic off-rate trends compared to the BIAS rankings, and of the five Fabs characterized, four show good agreement (~2-fold) of predicted *k*_off_ values compared to biochemically characterized *k*_off’_s.

A fair limitation of RAPID biopanning, particularly where stringent fluorescent activated sorting was needed, is that the binders identified from this method can yield lower expression levels as shown with SP1-B3 or be poorer growers. Expression of these binders in full-length IgG formats in mammalian cells can help overcome low expression yields in bacteria, which is a widely employed solution in improving Ab expression. As an example, SP1-B3 expressed in an IgG format shows a 220-fold increase in expression (33 mg/L culture) compared to its Fab counterpart. Although the current study presents three successful RAPID biopanning campaigns, it is important to note that the effectiveness of RAPID biopanning can still be influenced by several factors, including library design and in vitro selection conditions. Particularly, as RAPID FACS captures a mixture of clones, exploring their exact compositions of binders through next-generation sequencing (NGS) studies can further expand our understanding of this platform and the most effective ways to apply it generally.

While the RAPID biopanning pipeline synergizes high-quality ph-Fab population isolation with rapid discriminatory screening of candidate clones, each step is highly modular and can be used independently or in combination with other existing protocols. For example, as is common with YSD coupled with FACS, iterative rounds of fluorescent-activated sorting could be performed for continuous enrichment of high-quality ph-Fab populations. Also, where functional assays are available for hit screening, functional screens can be used in place of BIAS, post-flow cytometry profiling, and/or fluorescent-activated sorting. Finally, BIAS can be used with different Ab formats (i.e., scFv, nanobodies, etc.) and different anti-tag secondary IgGs as well.

In conclusion, we demonstrate that RAPID biopanning couples efficiency with precision to allow for a more selective biopanning campaign to identify rare high-affinity candidate clones, particularly for challenging targets.

## Methods

### Standard biopanning

Standard biopanning with magnetic beads was performed as previously described^20,21^. Briefly, a human naïve B-cell phage-displayed Fab library (diversity 4.1 × 10^10^) was used against CHIP and CS3D. Biotinylated Ags were immobilized to magnetic streptavidin beads (Dynabeads M-270 Streptavidin), and subsequently the ph-Fab library was added. For the CHIP campaign, 25 mM HEPES, 50 mM KCl was used for the binding, and 25 mM HEPES, 50 mM KCl, 0.05% Tween-20 was used for washing. For the CS3D campaign, 20 mM Tris-HCl, 150 mM NaCl, 0.05% NP-40, 1 mM EDTA was used for the binding, and both PBS and PBS-T (0.05% Tween-20) was used for washing. Both campaigns introduced negative selections against magnetic beads with no Ag starting at round 2. Individual clones were selected and screened with either Dot blots, ELISAs, and BIAS and subsequent hits were sequenced. Plasmids containing unique sequences of Fabs were used to transform BL21(DE3) *E. coli* cells for further biochemical analysis of Fab.

### Phage preparation

Fab-displayed phage were first amplified and prepared using standard methods. Briefly, 50 ml cultures (2xYT, 2% glucose, 100 μg/ml Ampicillin) of phagemid containing *E. coli* cells were incubated at 37 °C with 200 rpm shaking until OD_600_ reached ~0.5. Subsequently, 10 ml of this culture was infected with M13KO7 helper phage at 10:1 helper phage to cell ratio. Culture was incubated at 37 °C for 30 min without shaking followed by 20 min with shaking at 200 rpm. Infected cells were collected by centrifugation and cells were resuspended with fresh media (2xYT 100 μg/ml Ampicillin, 50 μg/ml Kanamycin). Cultures were grown overnight, and amplified phage were isolated by adding PEG 6000/2.5 M NaCl phage to the supernatant of the overnighted culture. Phage yield was analyzed by taking OD_268_ measurements.

### Phage labeling with NHS-FITC

NHS-Fluorescein (Thermo Scientific) was prepared in DMSO (1 mg/ml final concentration). For the standard protocol labeling reaction consisted of the following: 50 μl of NHS-FITC (1 mg/ ml) stock 500 μl of Phage (OD_268_ = 1, final), and 40 μl of Borate buffer (0.67 M). Previous studies have shown that NHS-esters, including NHS-FITC, can efficiently label M13 phage via the N-termini and lysine residues of the pVIII coat proteins^22,23^. Labeling reaction was incubated in RT for 1 hr in the dark. PEG 6000/2.5 M NaCl was added to the reaction to precipitate phage and incubated on ice for 15 min. Precipitated phage were collected by centrifugation (max speed for 5 min) and supernatant was removed. Pellets were resuspended in PBS and process was repeated. Phage were washed and precipitated three times total. Samples were immediately used in subsequent experiments. A plate reader (BioTek Synergy H4 plate reader) was used for fluorescent measurements and optical density of phage and FITC were analyzed using nanodrop. Phage labeling was aimed to maximize fluorescent signal per phage while maintaining solubility to achieve maximum dynamic range of fluorescent distribution. Labeling conditions were optimized using M13 helper phage. Normalized fluorescence shows an increase in relation to the number of FITC conjugated per phage, (Supplementary Fig 5a). Further labeling caused precipitation and was not further investigated. The washing and the reaction incubation time was also optimized (Supplementary Fig 5b, c) where a final reaction time of 1 h and a total of three washes determined to be optimal.

### Flow cytometry and fluorescent-activated sorting

All experiments of flow cytometry biopanning profiling and fluorescent activated sorting were done with biotinylated Ags immobilized with streptavidin-coated beads. SPHERO^TM^ Streptavidin Polystyrene particles, (3.0–3.9 μm) were used to immobilize Ag, and 100 μl of bead slurry was used against ph-Fab (OD_268_ = 0.1, 1 ml total volume). First, the beads were blocked with 2% BSA buffer for 1 h. Beads were then washed by resuspending beads in 1% BSA-buffer and subsequently centrifuging the beads (7k rpm for 2 min) to remove supernatant. This process was repeated three times. Biotinylated Ag was then added to beads at 1% BSA final concentration and incubated for one hour. Meanwhile, labeled phage were blocked in 1% BSA PBS for one hr. After Ag-immobilized beads were washed, blocked phage was added to the beads and incubated. After the binding phase, beads were washed 1 time with 1%BSA-buffer and passed through a 40−μm cell strainer. Subsequently, flow cytometry analysis or fluorescent-activated sorting was performed. Details of the gating strategy is listed in supplementary fig. 8. All sorting was performed on a BDFACS Aria II and all flow cytometry analysis were performed on a benchtop Beckman Cytoflex Analyzer or BD FACSCaliber machine. Bead populations were gated with FSC and SSC parameters and later only singlet populations were analyzed by gating linear FSA and FSW. Of singlet population of beads, histogram analysis was done with FITC. For fluorescent-activated sorting, correct percentage of events were sorted. Sorted beads were used to infect fresh TG1 cells (OD_600_ ~ 0.7) and cells were plate for picking individual clones.

### 96-well Periplasmic extract preparation

Single colony clones were picked and inoculated into 2xYT media containing 2% glucose and 100 mg/ml Ampicillin in round bottom 96 well plates (150 ml of media per well). Cultures were grown overnight at 37 °C with 200 rpm shaking. The following day 96 well cultures were inoculated (12 μl per well) into 96 well deep plates containing 2xYT media with 0.1% glucose and 100 μg/ml Ampicillin (1200 μl of media per well) and grown for 4–6 h until culture is turbid. Fab expression was induced with 300 μl of 2xYT, ampicillin mg/ml and 5 mM IPTG and were left overnight at 30 °C with 200 rpm shaking. Periplasmic extracts were collected by osmotic shock. Briefly, cells from overnight cultures were collected by centrifugation at 2000 g for 25 min and 375 μl of ice-cold TES buffer (200 mM Tris-HCl, 500 mM Sucrose, 0.5 mM EDTA, pH = 8) was added directly into each well and incubated with shaking at RT. Subsequently, 1125 μl of ice-cold water was added in each well and mixed thoroughly. The periplasmic fraction (supernatant) was collected by centrifugation at 2000 g for 25 min and stored at −20 °C for future experiments.

### Dot blots

From 96-well periplasmic extracts, 2-3 μl was applied to nitrocellulose membranes. After ~10 min, the membrane was blocked with 2%-TBS-milk and gently rocked at RT. After 1 h, membrane was gently rinsed with TBS-T (0.05% Tween-20) and 1%TBS-Milk with anti-myc HRP(9E10) (1:5000 dilution) was added and incubated for 1 hr. Washes were performed with TBS-T (x2) and TBS (x2), and 1 ml of Immobilon Forte Western HRP substrate was added for imaging on a ChmiDoc MP imaging system

### Biolayer interferometry (BLI)

All buffers were filter sterilized with 0.22 mm filters prior to preparing samples. Black 384 well microplates were used to set up BLI plates and streptavidin tips were purchased by Sartorius. Kinetic constants of Fabs were determined by Octet RED384 system with continuous shaking at 1000 rpm in RT. Prior to the experiment, biological tips (model name) were presoaked in buffer to allow for equilibration for 1 h. Data were analyzed using 1:1 interaction model on the ForteBio data analysis software 12.0

### Biolayer interferometry antibody screen (BIAS)

The instrument and reagent setup was identical to that of standard BLI. Anti-myc Ab (9E10) (Merk) was used for the Assoc-2. BIAS was performed on For the control experiments, BLI was run in following method: Baseline (1 min), Load, Baseline (1 min), Assoc-1 (10 min), Assoc-2 (10 min), Dissoc (10 min), and for CHIP, Gαq, and CS3D BIAS runs, Assoc-1 (3 min), Assoc-2 (2 min 30 sec), and Dissoc (3 min) was used as the final protocol. Dependent on the Ag that was used, loading step was adjusted where loading was immediately terminated once rate of loading changed to allow for equal distribution of Ag on tip. Raw data files of the run(s) were then used in the BATCH to rank order candidate hits according to their predicted dissociation rates. The true Assoc-2 slope of the PPE spiked sample without 9E10 calculated by BATCH was further used as the threshold value for determining hits (Supplementary Table 1).

### BIAS Algorithm Triaging Confirmed Hits (BATCH) development

A BIAS function was developed to effectively process raw BLI sensogram data, to categorize and rank order candidate hits according to their kinetic properties. A brief schematic of the categorization, ranking system, and outputs is listed in Supplementary Fig 3. BATCH will process all raw BLI data files, a user-created methods file, and a user-created thresholds file. Due to buffer mismatches occurring during the BIAS runs, each step is trimmed at the beginning and end to avoid erroneous noise. Briefly, the Assoc-1 and Dissoc steps are fitted as a one-phase exponential association and one-phase exponential decay respectively. To account for continued Fab binding during the Assoc-2 step, the one-phase exponential association fit of the Assoc-1 step is extrapolated through Assoc-2 as the extrapolated association-1 curve. The true Assoc-2 slope only accounts for the anti-myc IgG signal contribution and is calculated by subtracting the extrapolated association curve by the raw Assoc-2 curve. The true Assoc-2 slope is fitted with a linear regression to quantify a significant signal shift. Importantly the true Assoc-2 slope distinguishes hits versus false positives. The thresholds for true Assoc-2 curve slope, true Assoc-2 *R*^2^, extrapolated Assoc-1 slope, and Dissoc *R*^2^ were all determined by the values obtained from the control experiments of P1A4 spiked in PPE (supplementary table 1). The Assoc-1 Total change threshold was determined by the control experiment where 25 nM of P1A4 was spiked in PPE, and BIAS k_off_ values were more than 2-fold less than the true value (supplementary table 2). BATCH outputs *R*^2^ values for all fits in each step which determines which category each clone is classified into based on a threshold value (Supplementary Fig 4). BATCH assumes a pseudo-first-order kinetic model for predicting *k*_off_s. By using the calculated *k*_off_s from the Dissoc step, BATCH will predict a range of *K*_d_ based on previous Craik lab Fab *k*_on_s and rank clones classified as hits. BATCH will generate two tables to provide a summary of results and a more detailed view of all processed data. To give the user more information on the reason behind classification of clones, the comment section provides more details (i.e., no dissociation, no association, *R*^2^ values too low, etc.). Threshold and *k*_on_ values can be changed according to the user’s experimental setup where secondary IgG or Ab format is different.

### Exceptions to the BIAS rankings

Clones that are flagged in the BIAS rankings indicate inaccuracies in *k*_off_ predictions. For these clones, the true association-2 slope can be a better predictor of binding, as exchange rates of Fab to Fab-9E10 complex during Assoc-2 are directly correlated to *k*_off_ of the Fab. Specifically, rapid exchange of Fab to Fab-9E10 indicate a high *k*_off_, which translate to a high true association-2 slope. Therefore, *k*_off_ is inversely proportional to the true association-2 slope. A simple guide to further distinguishing flagged BIAS results is shown in Supplementary Fig 6.

### Fab expression

Freshly transformed BL21(DE3) *E. coli* single colony clones were picked and inoculated into 50 ml of 2xYT media containing 2% glucose and 100 mg/ml Ampicillin. Starter cultures were grown overnight at 37 °C with 200 rpm shaking. The next day, the starter culture was inoculated to 1 L of 2xYT media with 0.1% glucose and 100 mg/ml Ampicillin (OD_600_ 0.05 final) and after incubation (37 °C with 200 rpm shaking), protein expression was induced with IPTG (1 mM final) at OD_600_ of 0.6 and continued to grow overnight (20 °C with 200 rpm shaking). Periplasmic extracts containing Fabs were collected by osmotic shock. Briefly, cells from overnight cultures were collected by centrifugation at 9000 *g* for 15 min and ~15 ml of ice-cold TES buffer was added and resuspended thoroughly to achieve a homogenous mixture. The mixture was then incubated with gentle shaking at 4 °C for 1 h. Subsequently, ~25 ml of ice-cold water was added and incubated with gentle shaking for 45 min. The periplasmic fraction (supernatant) was collected by centrifugation at 10,000 g for 30 min and loaded to Ni-NTA resin for affinity purification. Purified Fabs were dialyzed against PBS (10 kDa MWCO) and size exclusion chromatography was performed to further purify the Fabs and remove any aggregates (AKTA autopurification system with a Superdex 200 10/300GL column). Fractions exhibiting correct size were pooled and Fab concentrations were determined by absorbance at 280 nm.

### Conversion of Fab into Full-Length IgG

The methods used here were described in detail in a previous study^20^. Briefly, the heavy and light chain regions of Fabs were cloned out from their respective phagemids and then individually cloned into a pTT5-SP-H1 mammalian expression vector through Gibson Assembly® (NEB #E2611) methods. The Gibson assembly product was transformed into NEB® 5α Competent E. Coli cells (NEB #C2987H) and plasmids were isolated using a ZymoPureII Plasmid Maxiprep Kit (Zymo Research #D4203) and confirmed through sequencing.

### Small-scale expression and purification of full-length IgG in mammalian cells

The expression protocol used here was based on the Expi293^TM^ Expression System (ThermoFisher #A14635). Expi293 suspension cells were seeded at a final density of 2.5 ×10^6^ cells/mL in a 6-well plate. The next day cells were diluted to a final density of 3 ×10^6^ cells/mL and a total of 1.0 μg/mL of plasmid DNA was transfected using ExpiFectamine^TM^ 293 Transfection Kit. Transfected cells were then incubated on an orbital shaker at 37 °C and 8% CO_2_ overnight and ExpiFectamine 293 Transfection Enhancer 1 and 2 were added to the cells. Secreted IgG was harvested 6 days post-transfection and the supernatant was incubated with Pierce^TM^ Protein G Plus Agarose (ThermoScientific #22851) resin at room temperature for 1 h. After washing with 10 column volumes of PBS, the IgG was eluted with 2 column volumes of 100 mM glycine, pH 2.5 and neutralized by 1 M Tris, pH 8.5. The eluted fractions were determined by SDS-PAGE gel and fractions containing pure IgG were collected and dialyzed against PBS buffer.

### Statistics and reproducibility

NHS-FITC phage labeling experiments were performed in triplicates and data were presented as mean ± SD. Flow cytometry and FACS data was analyzed by FlowJo 10.9.0 from at least 10,000 events. The statistical significance between the BIAS off-rates of sorted and unsorted populations were determined by A two-way ANOVA test on Prism (Graphpad).

### Reporting summary

Further information on research design is available in the Nature Portfolio Reporting Summary linked to this article.

### Supplementary information

Peer Review File

Supplementary Information

Description of Additional Supplementary Files

Supplementary Data 1

Reporting Summary

## Data Availability

Source data for the main figures are available as Supplementary data 1 and any remaining information can be obtained from the corresponding author upon reasonable request.

## Code availability

The code can be made available upon request under an MTA.
