# Supplementary material for: Rare antibody phage isolation and discrimination (RAPID) biopanning enables identification of high-affinity antibodies against challenging targets
Source: Commun Biol. 2023 Oct 12;6:1036. doi: 10.1038/s42003-023-05390-0 (PMC10570357; doi:10.1038/s42003-023-05390-0)
Supplement: Supplementary file 2 — Supplementary Information [file 42003_2023_5390_MOESM2_ESM.pdf]

Supplementary Information: Rare Antibody Phage Isolation and Discrimination (RAPID) biopanning enables identification of high-affinity antibodies against challenging targets

Dong hee Chung[1], Sophie Kong[1], Nicholas J. Young[1], Shih-Wei Chuo[1], Jamie V. Shiah[2], Emily J. Connelly[3], Peter J. Rohweder[1], Alexandra Born[1], Aashish Manglik[1], Jennifer R. Grandis[2], Daniel E. Johnson[2], Charles S. Craik[1,3#]

1 Department of Pharmaceutical Chemistry, University of California San Francisco, San Francisco, CA 94158, USA.
2 Department of Otolaryngology – Head and Neck Surgery, University of California San Francisco, CA 94158, USA.
3 The Pharmaceutical Sciences and Pharmacogenomics Graduate Program, University of California San Francisco, San Francisco, CA 94158, USA.
#Corresponding Author, charles.craik@ucsf.edu

Supplementary Figures

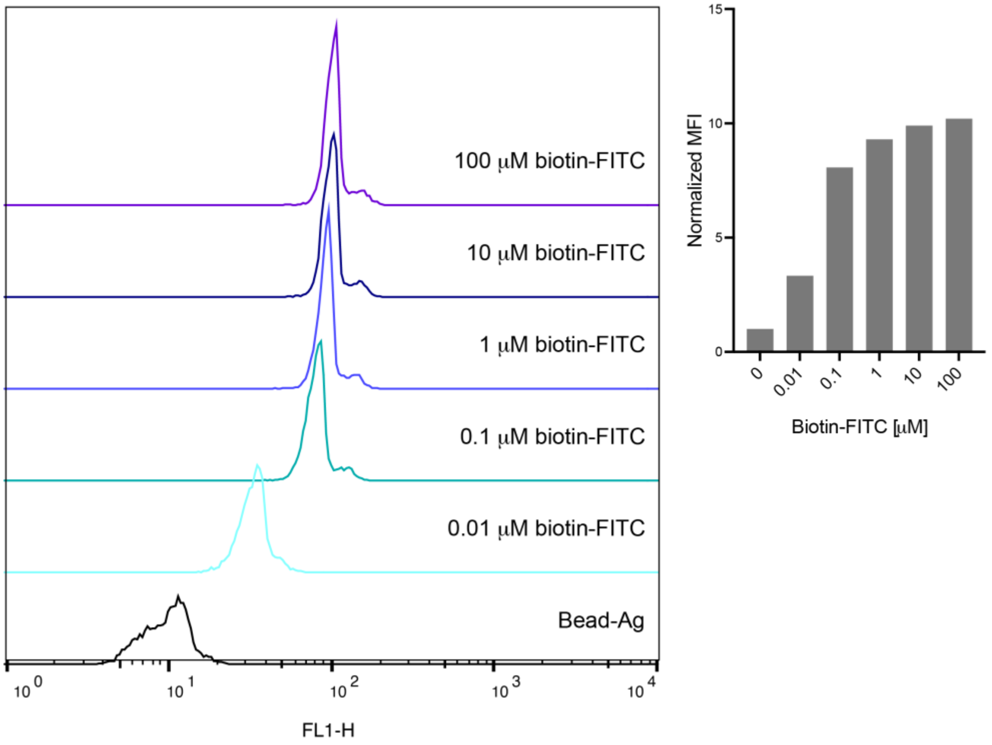

Supplementary figure 1. Increasing concentrations of biotin-FITC to streptavidin beads . A maximal distribution shift is observed starting at 1 μM biotin-FITC immobilization with no overlap to the negative control with no phage.

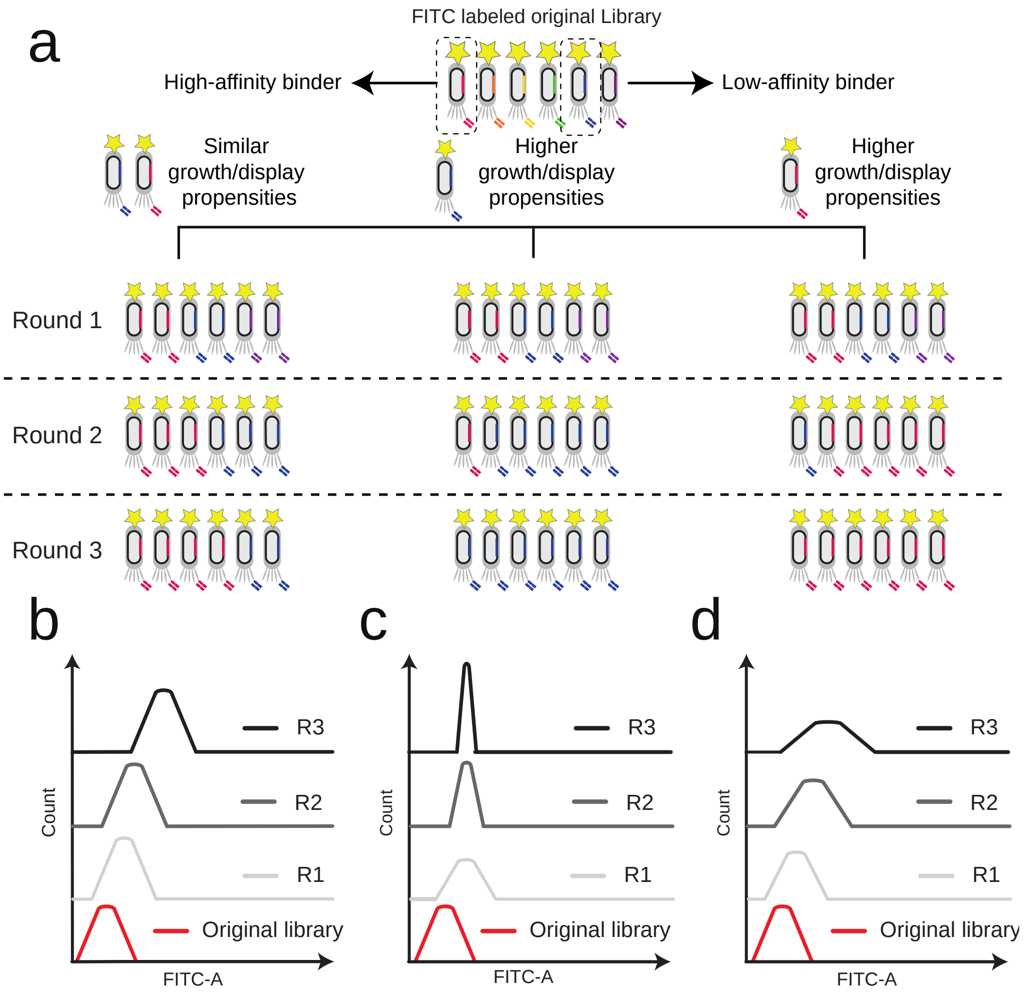

Supplementary figure 2. RAPID Flow cytometry enrichment profiling with fluorescent labeled ph-Fab libraries. (a) Depending on the growth/display propensities of phage, and the differences between the affinity of higher and lower affinity ph-Fabs, iterative rounds of biopanning result in different relative populations of ph-Fab binders. (b) Where growth/display levels are similar between ph-Fab library members, flow distributions show increase in normalized MFI while the standard deviation is similar (SD). (c) Where high-affinity ph-Fabs exhibit inferior growth/display propensities, MFI increase is halted prematurely, and SD decreases as weaker binders are more prevalent. (d) Where high-affinity ph-Fabs also exhibit superior growth/display propensities, an increasing in MFI and an increase in SD is observed.

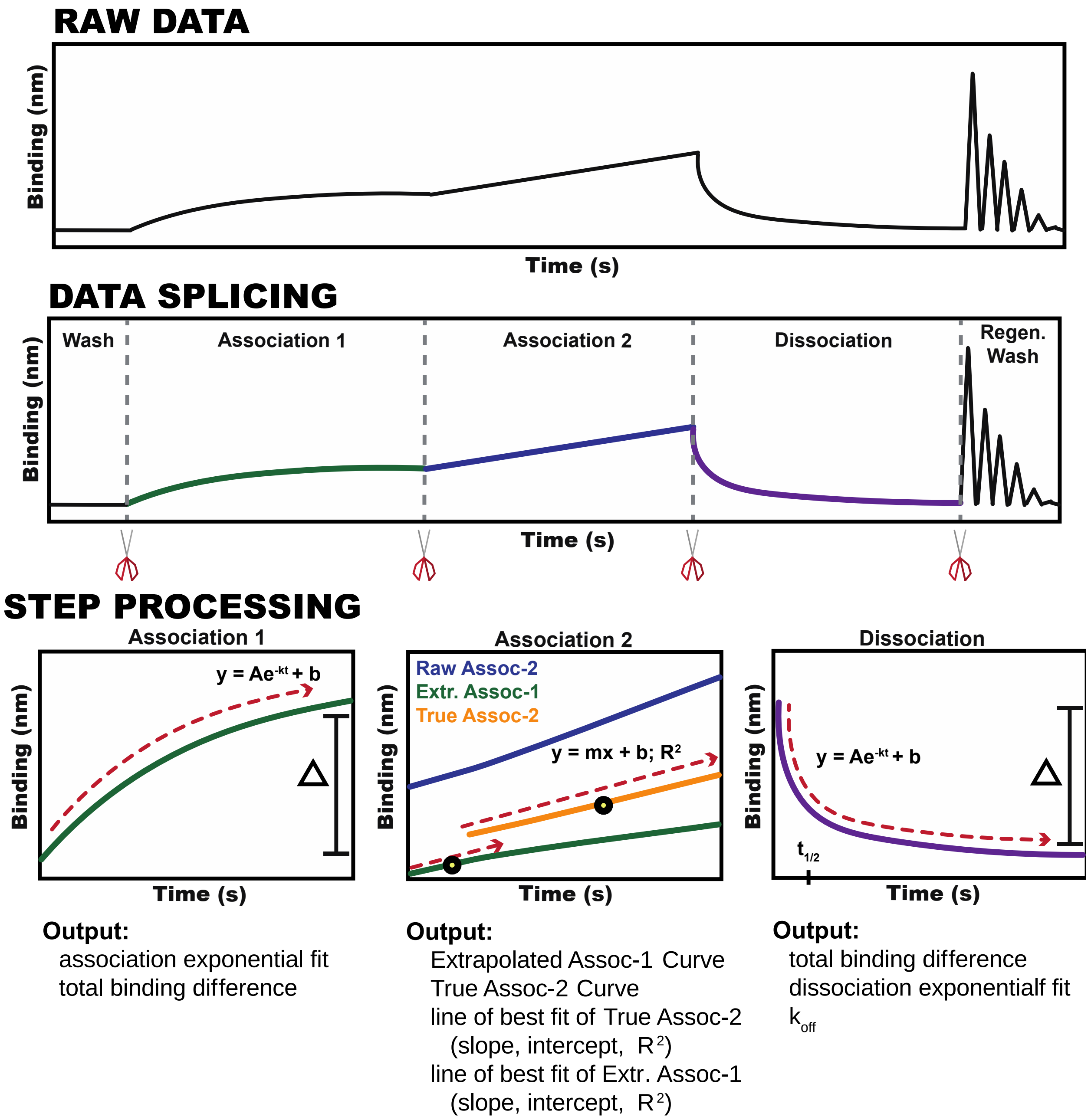

Supplementary figure 3. BATCH categorization, ranking, and output scheme.

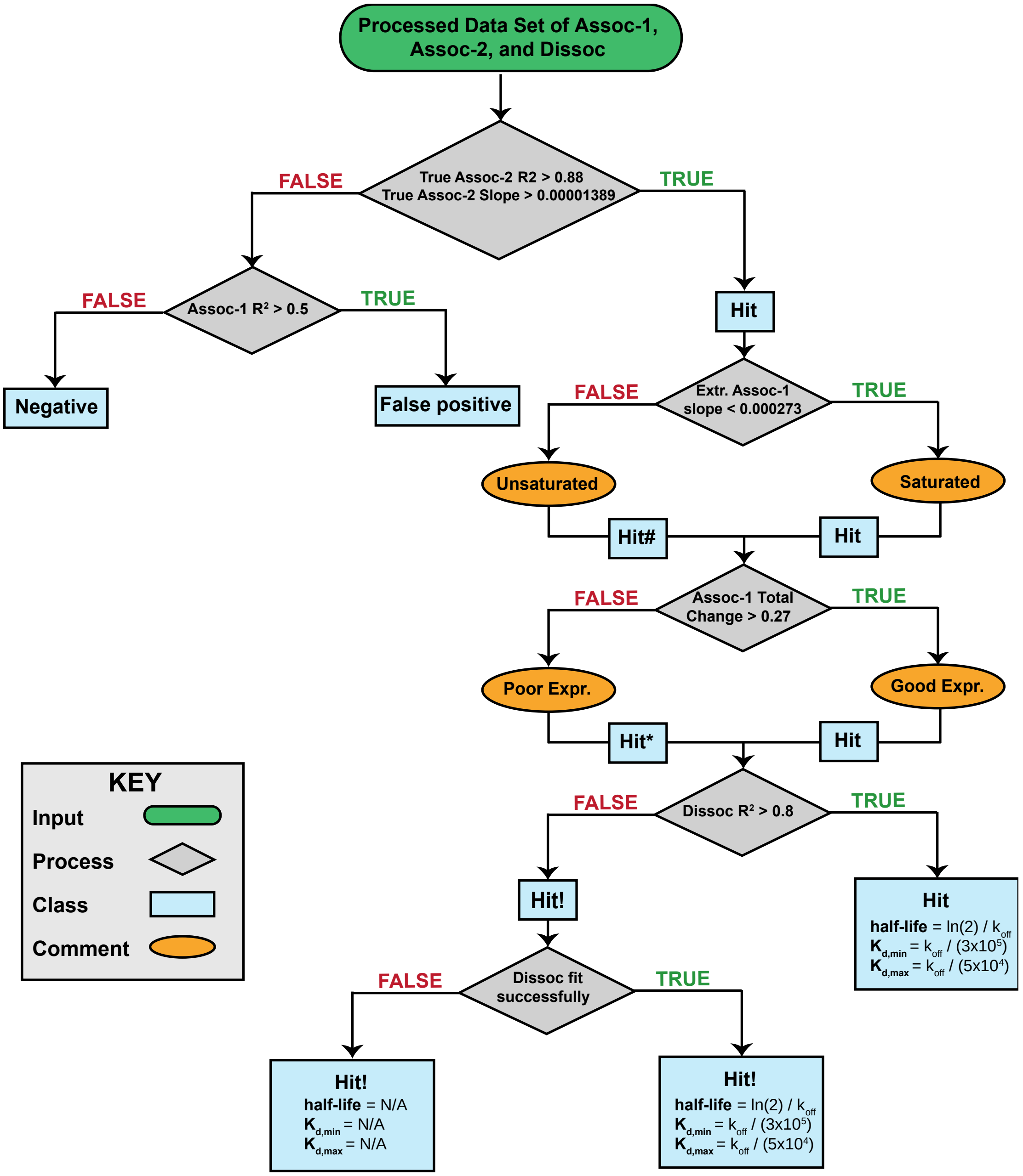

Supplementary figure 4. BATCH algorithm scheme. The thresholds for "true Assoc-2 curve slope", "true Assoc-2 R^2", "extrapolated Assoc-1 slope", and "Dissoc R^2" were all determined by the values obtained from the control experiments of P1A4 spiked in PPE. The "Assoc-1 Total change" threshold was determined by the control experiment where 25 nM of P1A4 was spiked in PPE, and BIAS k_{off} values were more than 2-fold less than the true value.

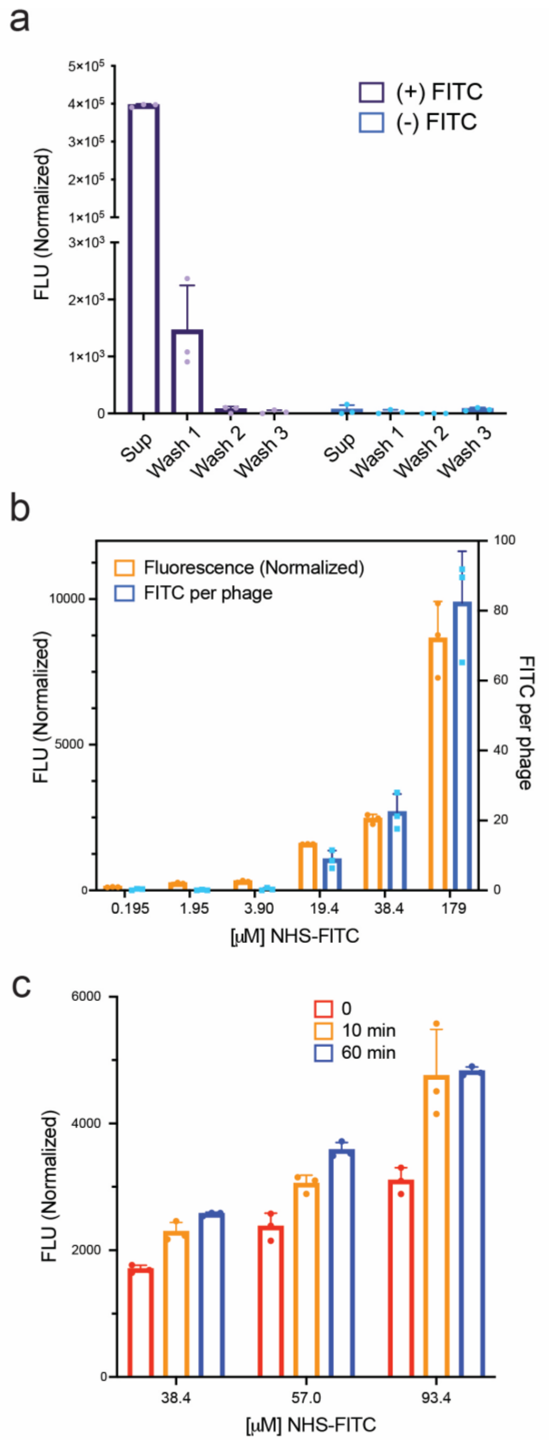

Supplementary figure 5. Optimization of phage labeling with NHS-FITC and M13 phage. experiments were performed in n=3 and data is presented as mean ± SD (a) Fluorescent signal is increased as FITC labelling concentration is increased FITC labeling. (b) Washing optimizations show three washing steps are sufficient for complete depletion of non-reacted NHS-FITC. (c) FITC labeling is saturated after 1 h reaction time.

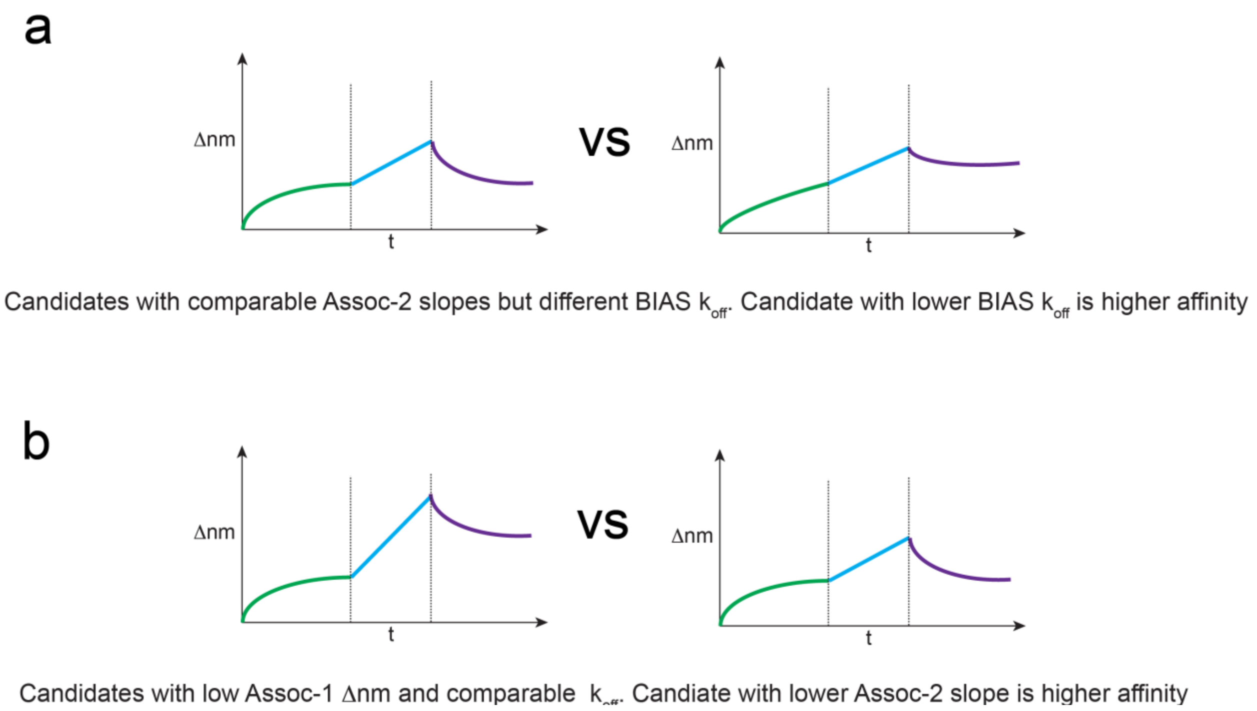

Supplementary figure 6. Simple guide to ranking flagged clones from BIAS screens. (a) For candidates that exhibit comparable Assoc -2 slopes but show different BIAS k_{off}s, the clone with lower k_{off} is the more promising binder. (b) For candidates that are flagged to not fully saturate or show very low expression levels, Assoc-2 curves are better indications of binding affinity than BIAS k_{off}. Roughly, lower true Assoc-2 slopes indicate higher affinity.

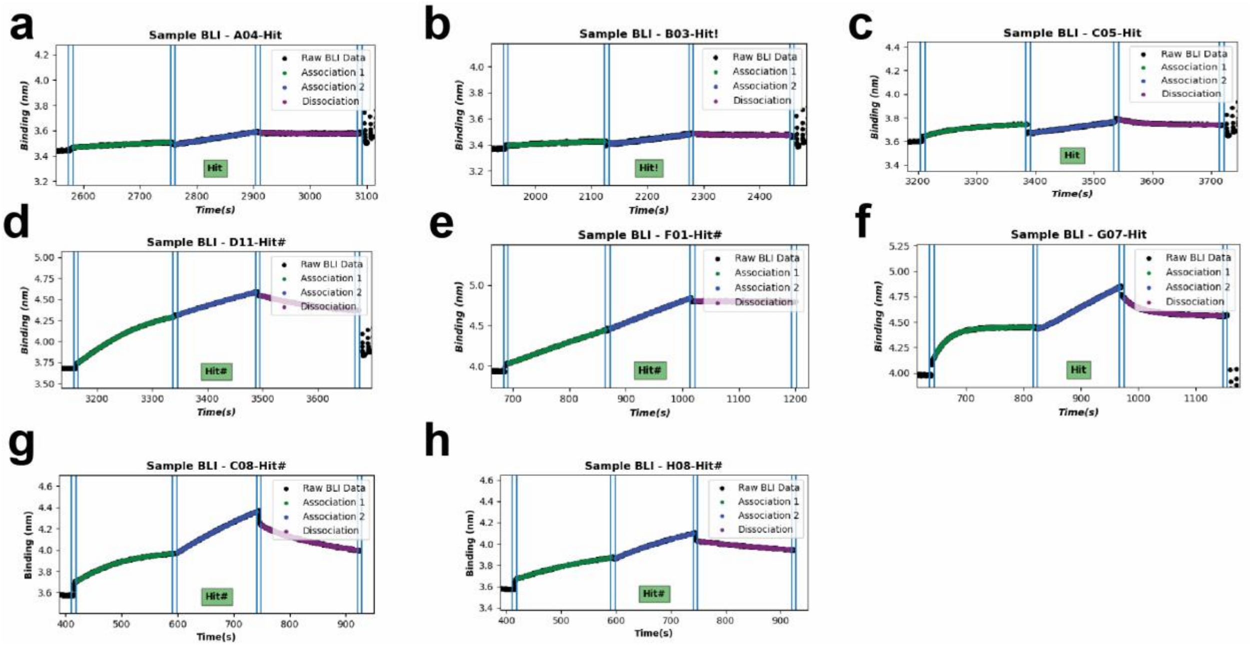

Supplementary figure 7. BIAS traces of CHIP (a)-(f) and Gαq hits from BATCH. (g)-(f). Assoc-1, Assoc-2, Dissoc steps are marked with green, blue, purple respectfully.

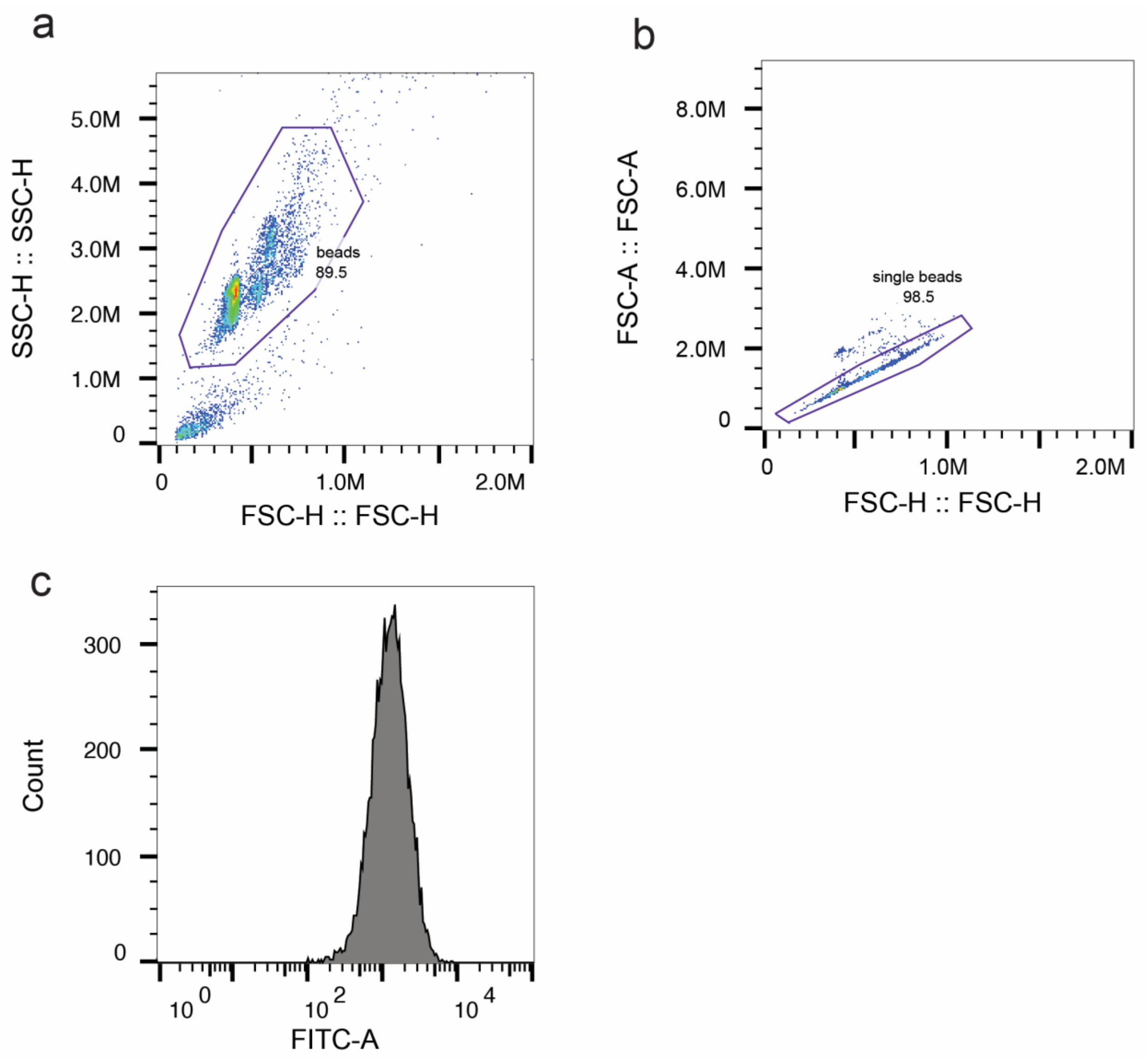

Supplementary figure 8. Gating strategy for RAPID Flow cytometry and FACS. (a) Bead populations were gated with FSC and SSC parameters. (b) Singlet Bead populations were gated with FSC and FSA. (c) Of singlet population of beads, histogram analysis was done with FITC.

Supplementary Tables

Supplementary Table 1. BIAS with pure P1A4 spiked in PBS and PPE.

	P1A4-9E10 (PBS)	P1A4-P1A4 (PBS)	P1A4-9E10 (PPE)	P1A4-P1A4 (PPE)
True Assoc-2 Slope (nm s^{-1})	7.1×10^{-4}	1.33×10^{-4}	3.94×10^{-4}	1.39×10^{-4}
BIAS k_{off} (s^{-1})	2.48×10^{-3}	2.58×10^{-3}	2.27×10^{-3}	2.33×10^{-3}

Supplementary Table 2. BIAS kinetic parameters (k_{off}, true Assoc-2 slope, and extrapolated assoc-2 linear slope) of multiple concentrations of P1A4 spiked in PPE.

P1A4 concentration	BIAS k_{off} (s^{-1})	True Assoc-2 Slope (nm s^{-1})	Extrapolated Assoc-2 Linear slope (nm s^{-1})
300 nM	2.28×10^{-3}	8.07×10^{-4}	4.79×10^{-6}
150 nM	2.38×10^{-3}	8.55×10^{-4}	2.12×10^{-5}
100 nM	1.70×10^{-3}	1.19×10^{-3}	8.75×10^{-5}
75 nM	1.74×10^{-3}	1.13×10^{-3}	1.34×10^{-4}
50 nM	1.39×10^{-3}	1.31×10^{-3}	2.73×10^{-4}
25 nM	4.50×10^{-4}	1.35×10^{-3}	6.86×10^{-4}
10 nM	6.28×10^{-5}	1.41×10^{-3}	9.64×10^{-4}

Supplementary Table 3. BIAS hit summary table for CHIP (a) and Gαq (b). Inaccurate k_{off} measurements are flagged (*-low expression #-unsaturuated Assoc-1, !-poor exponential fit for Dissoc). Kd min and max are estimated based on general k_{on} of previously discovered Craik lab Fabs (5×10^4 - 3×10^5 $M^{-1}s^{-1}$)

a

Rank	Sample ID	Classification	k off (s^{-1})	Kd min (M)	Kd max (M)
1	F1	Hit#	9.40×10^{-6}	3.13×10^{-11}	1.88×10^{-10}
2	F2	Hit#	8.17×10^{-4}	2.72×10^{-9}	1.63×10^{-8}
3	D11	Hit#	7.33×10^{-3}	2.44×10^{-8}	1.47×10^{-7}
4	C9	Hit*	1.47×10^{-2}	4.90×10^{-8}	2.94×10^{-7}
5	D2	Hit#	1.87×10^{-2}	6.23×10^{-8}	3.74×10^{-7}
6	A4	Hit	2.28×10^{-2}	7.61×10^{-8}	4.57×10^{-7}
7	E4	Hit!	2.30×10^{-2}	7.67×10^{-8}	4.60×10^{-7}
8	C5	Hit	2.60×10^{-2}	8.67×10^{-8}	5.20×10^{-7}
9	G7	Hit	2.92×10^{-2}	9.75×10^{-8}	5.85×10^{-7}
10	B3	Hit!	3.37×10^{-2}	1.12×10^{-7}	6.74×10^{-7}
11	H11	Hit	4.50×10^{-2}	1.50×10^{-7}	9.01×10^{-7}
12	G4	Hit*!	5.44×10^{-2}	1.81×10^{-7}	1.09×10^{-6}
13	B10	Hit*!	--	--	--
14	B11	Hit!	--	--	--
15	D3	Hit#!	--	--	--
16	D8	Hit*!	--	--	--
17	E2	Hit#!	--	--	--
18	F3	Hit!	--	--	--

b

Rank	Sample ID	Classification	k off (s^{-1})	Kd min (M)	Kd max (M)
1	H8	Hit#	2.99×10^{-3}	9.97×10^{-9}	5.98×10^{-8}
2	B9	Hit#	4.05×10^{-3}	1.35×10^{-8}	8.10×10^{-8}
3	D8	Hit#	4.17×10^{-3}	1.39×10^{-8}	8.33×10^{-8}
4	C10	Hit#	4.28×10^{-3}	1.43×10^{-8}	8.55×10^{-8}
5	E8	Hit#	4.30×10^{-3}	1.43×10^{-8}	8.59×10^{-8}
6	G9	Hit#	4.33×10^{-3}	1.44×10^{-8}	8.66×10^{-8}
7	E7	Hit#	4.40×10^{-3}	1.47×10^{-8}	8.80×10^{-8}
8	B8	Hit#	4.54×10^{-3}	1.51×10^{-8}	9.07×10^{-8}
9	E9	Hit#	4.55×10^{-3}	1.52×10^{-8}	9.09×10^{-8}
10	E5	Hit#	4.76×10^{-3}	1.59×10^{-8}	9.52×10^{-8}
11	C4	Hit#	5.17×10^{-3}	1.72×10^{-8}	1.03×10^{-7}

12	E1	Hit#	5.50×10^{-3}	1.83×10^{-8}	1.10×10^{-7}
13	C8	Hit#	7.79×10^{-3}	2.60×10^{-8}	1.56×10^{-7}
14	C6	Hit#	7.95×10^{-3}	2.65×10^{-8}	1.59×10^{-7}
15	D12	Hit	7.98×10^{-3}	2.66×10^{-8}	1.60×10^{-7}
16	A10	Hit#	8.10×10^{-3}	2.70×10^{-8}	1.62×10^{-7}
17	E4	Hit	8.80×10^{-3}	2.93×10^{-8}	1.76×10^{-7}
18	D7	Hit	9.44×10^{-3}	3.15×10^{-8}	1.89×10^{-7}
19	F1	Hit*	1.01×10^{-2}	3.37×10^{-8}	2.02×10^{-7}
20	A7	Hit*	1.05×10^{-2}	3.50×10^{-8}	2.10×10^{-7}
21	E10	Hit	1.22×10^{-2}	4.05×10^{-8}	2.43×10^{-7}
22	D11	Hit*	1.22×10^{-2}	4.07×10^{-8}	2.44×10^{-7}
23	E11	Hit	1.28×10^{-2}	4.25×10^{-8}	2.55×10^{-7}
24	D5	Hit*	1.31×10^{-2}	4.38×10^{-8}	2.63×10^{-7}
25	E6	Hit*	1.41×10^{-2}	4.68×10^{-8}	2.81×10^{-7}
26	A11	Hit*	1.52×10^{-2}	5.07×10^{-8}	3.04×10^{-7}
27	E12	Hit*	1.84×10^{-2}	6.13×10^{-8}	3.68×10^{-7}

Supplementary Table 4. k_{off} (measured at 2μM) and recombinant expression yields *(E. coli)* of Fabs from CS3D biopanning.

Fab	3C8	3B11	3B12	2C11	3B12	4E4	SP1-B3
k_{off} (s^{-1})	2.77×10^{-1}	2.05×10^{-1}	7.64×10^{-2}	4.19×10^{-1}	3.7×10^{-1}	1.11×10^{-1}	2.11×10^{-2}
Expression (mg L^{-1} culture)	2.7	2.4	1.7	0.9	1.4	0.3	0.1

Supplementary Table 5. BIAS of randomly chosen clones from Round 4-6 of panning and Round 4 PhAB sorting.

	Round 4	Round 5	Round 6	Round 4 sorted (top 4%)	Round 4 sorted (bottom 96%)
Hits (#,*)	69	84	77	37	69
False positive	17	11	17	106	21
Negative	9	0	1	47	5
Total	95	95	95	190	95

Supplementary Table 6. Top 10 BIAS ranked candidate clones from standard biopanning Round 4-6 and their k_{off}s measured by BLI at 2 μM Fab concentration.

BIAS Rank	Round 2 Clone ID	koff (s^{-1})	Round 3 Clone ID	koff (s^{-1})	Round 4 Clone ID	koff (s^{-1})
1	2A12	7.64×10^{-2}	3G3	2.05×10^{-1}	4G1	7.64×10^{-2}
2	2C6	2.05×10^{-1}	3F2	2.05×10^{-1}	4D12	7.64×10^{-2}
3	2C12	7.64×10^{-2}	3F12	7.64×10^{-2}	4H1	7.64×10^{-2}
4	2B12	3.70×10^{-1}	3C3	2.05×10^{-1}	4C3	7.64×10^{-2}
5	2D6	3.70×10^{-1}	3B11	2.05×10^{-1}	4B1	7.64×10^{-2}
6	2C9	7.64×10^{-2}	3C12	7.64×10^{-2}	4F1	7.64×10^{-2}
7	2B5	4.19×10^{-1}	3C8	2.77×10^{-1}	4B2	7.64×10^{-2}
8	2A7	2.05×10^{-1}	3E12	7.64×10^{-2}	4C2	7.64×10^{-2}
9	2C11	4.19×10^{-1}	3C9	2.77×10^{-1}	4E1	7.64×10^{-2}
10	2B2	4.19×10^{-1}	3B12	7.64×10^{-2}	4E2	7.64×10^{-2}
