# Supplementary material for: Rare antibody phage isolation and discrimination (RAPID) biopanning enables identification of high-affinity antibodies against challenging targets
Source: Commun Biol. 2023 Oct 12;6:1036. doi: 10.1038/s42003-023-05390-0 (PMC10570357; doi:10.1038/s42003-023-05390-0)
Supplement: Supplementary file 3 — Description of Additional Supplementary Files [file 42003_2023_5390_MOESM3_ESM.pdf]

Description of Additional Supplementary Files

File name: Supplementary Data 1
Description: Source data for the main figures.
