# Supplementary material for: Rare antibody phage isolation and discrimination (RAPID) biopanning enables identification of high-affinity antibodies against challenging targets
Source: Commun Biol. 2023 Oct 12;6:1036. doi: 10.1038/s42003-023-05390-0 (PMC10570357; doi:10.1038/s42003-023-05390-0)
Supplement: Supplementary file 5 — Reporting Summary [file 42003_2023_5390_MOESM5_ESM.pdf]

nature portfolio

Corresponding author(s): Charles S. Craik

Last updated by author(s): Sep 6, 2023

Reporting Summary

Nature Portfolio wishes to improve the reproducibility of the work that we publish. This form provides structure for consistency and transparency in reporting. For further information on Nature Portfolio policies, see our Editorial Policies and the Editorial Policy Checklist.

Statistics

For all statistical analyses, confirm that the following items are present in the figure legend, table legend, main text, or Methods section.

n/a	Confirmed	
☒	☐	The exact sample size (*n*) for each experimental group/condition, given as a discrete number and unit of measurement
☐	☒	A statement on whether measurements were taken from distinct samples or whether the same sample was measured repeatedly
☒	☐	The statistical test(s) used AND whether they are one- or two-sided<br>*Only common tests should be described solely by name; describe more complex techniques in the Methods section.*
☒	☐	A description of all covariates tested
☐	☒	A description of any assumptions or corrections, such as tests of normality and adjustment for multiple comparisons
☐	☒	A full description of the statistical parameters including central tendency (e.g. means) or other basic estimates (e.g. regression coefficient) AND variation (e.g. standard deviation) or associated estimates of uncertainty (e.g. confidence intervals)
☐	☒	For null hypothesis testing, the test statistic (e.g. *F*, *t*, *r*) with confidence intervals, effect sizes, degrees of freedom and *P* value noted<br>*Give P values as exact values whenever suitable.*
☒	☐	For Bayesian analysis, information on the choice of priors and Markov chain Monte Carlo settings
☒	☐	For hierarchical and complex designs, identification of the appropriate level for tests and full reporting of outcomes
☒	☐	Estimates of effect sizes (e.g. Cohen's *d*, Pearson's *r*), indicating how they were calculated

Our web collection on statistics for biologists contains articles on many of the points above.

Software and code

Policy information about availability of computer code

Data collection	BLI data was collected by Octet BLI Discovery 12.2. Flow cytometry data was collected by CytExpert version 2.5.0.77 software. Fluorescent activated sorting data was collected by. Plate reader data was collected by Gen5 3.10 software.
Data analysis	FlowJo 10.8.1 was used to analyze flow cytometry and Fluorescent activated sorting data. BLI data was analyzed with ForteBio Data Analysis 12.0.2.3. Plate reader data was exported and analyzed separately using Prism 9 software. Custom code (BATCH) was used to analyze BLI data, which is reported with the manuscript.

For manuscripts utilizing custom algorithms or software that are central to the research but not yet described in published literature, software must be made available to editors and reviewers. We strongly encourage code deposition in a community repository (e.g. GitHub). See the Nature Portfolio guidelines for submitting code & software for further information.

Data

Policy information about availability of data

All manuscripts must include a data availability statement. This statement should provide the following information, where applicable:

- Accession codes, unique identifiers, or web links for publicly available datasets
- A description of any restrictions on data availability
- For clinical datasets or third party data, please ensure that the statement adheres to our policy

The datasets generated during and/or analysed during the current study are available from the corresponding author on reasonable request.

Human research participants

Policy information about studies involving human research participants and Sex and Gender in Research.

Reporting on sex and gender	n/a
Population characteristics	n/a
Recruitment	n/a
Ethics oversight	n/a

Note that full information on the approval of the study protocol must also be provided in the manuscript.

Field-specific reporting

Please select the one below that is the best fit for your research. If you are not sure, read the appropriate sections before making your selection.

☒ Life sciences ☐ Behavioural & social sciences ☐ Ecological, evolutionary & environmental sciences

For a reference copy of the document with all sections, see nature.com/documents/nr-reporting-summary-flat.pdf

Life sciences study design

All studies must disclose on these points even when the disclosure is negative.

Sample size	Sample sizes and requisite statistical tests are indicated for each experiment in the methods section.
Data exclusions	No data was excluded
Replication	Technical replicates and statistical tests are reported where appropriate. All experiments to confirm repeatability were successful.
Randomization	Randomization of samples was not relevant to this study. Experimental and control samples were validated biochemically as appropriate.
Blinding	Blinding was not applicable to this study.

Reporting for specific materials, systems and methods

We require information from authors about some types of materials, experimental systems and methods used in many studies. Here, indicate whether each material, system or method listed is relevant to your study. If you are not sure if a list item applies to your research, read the appropriate section before selecting a response.

Materials & experimental systems

n/a	Involved in the study	
☐	☒	Antibodies
☐	☒	Eukaryotic cell lines
☒	☐	Palaeontology and archaeology
☒	☐	Animals and other organisms
☒	☐	Clinical data
☒	☐	Dual use research of concern

Methods

n/a	Involved in the study	
☒	☐	ChIP-seq
☐	☒	Flow cytometry
☒	☐	MRI-based neuroimaging

Antibodies

Antibodies used	Anti-c-myc-Peroxidase (9E10), Supplier: Roche, Catalog No. 11814150001. Additional antibodies were identified during the course of this study. They have been reported in the manuscript.
Validation	For the BIAS control experiments, only the samples containing a myc tag shows an additional secondary association curve in the Assoc-2 step. 9E10 is highly specific where a single amino acid change has been reported to disrupt myc-9E10 binding. Provided reference : Hilpert, K. et al. Anti-c-myc antibody 9E10: epitope key positions and variability characterized using peptide spot synthesis on cellulose. Protein Eng. Des. Sel. 14, 803–806 (2001).

Eukaryotic cell lines

Policy information about cell lines and Sex and Gender in Research

Cell line source(s)	Expi293 suspension cell (ThermoFisher #A14635)
Authentication	Cell lines were used directly as provided by manufacturer without additional authentication.
Mycoplasma contamination	Expi293 suspension cells were tested by manufacturer prior to use. No additional testing was done.
Commonly misidentified lines (See ICLAC register)	*Name any commonly misidentified cell lines used in the study and provide a rationale for their use.*

Flow Cytometry

Plots

Confirm that:

☒ The axis labels state the marker and fluorochrome used (e.g. CD4-FITC).

☒ The axis scales are clearly visible. Include numbers along axes only for bottom left plot of group (a 'group' is an analysis of identical markers).

☐ All plots are contour plots with outliers or pseudocolor plots.

☒ A numerical value for number of cells or percentage (with statistics) is provided.

Methodology

Sample preparation	No cells were sorted in this manuscript. Streptavidin polystyrene beads were sorted with fluorescently labeled phage samples. Source information and preparation of the phage are reported in the methods section.
Instrument	Beckman Cytoflex Analyzer, BDFACSCaliber, BDFACS Aria II
Software	FlowJo 10.8.1
Cell population abundance	Sorted samples were isolated from homogenous starting material. Over 95% of sorted beads were determined to be singlets as detailed below.
Gating strategy	Bead populations were gated with FSC and SSC parameters. Singlet Bead populations were gated with FSC and FSA. Of singlet population of beads, histogram analysis was done with FITC.

☒ Tick this box to confirm that a figure exemplifying the gating strategy is provided in the Supplementary Information.
